# 3DeeCellTracker, a deep learning-based pipeline for segmenting and tracking cells in 3D time lapse images

**DOI:** 10.7554/eLife.59187

**Published:** 2021-03-30

**Authors:** Chentao Wen, Takuya Miura, Venkatakaushik Voleti, Kazushi Yamaguchi, Motosuke Tsutsumi, Kei Yamamoto, Kohei Otomo, Yukako Fujie, Takayuki Teramoto, Takeshi Ishihara, Kazuhiro Aoki, Tomomi Nemoto, Elizabeth MC Hillman, Koutarou D Kimura

**Affiliations:** 1 Graduate School of Science, Nagoya City UniversityNagoyaJapan; 2 Department of Biological Sciences, Graduate School of Science, Osaka UniversityToyonakaJapan; 3 Departments of Biomedical Engineering and Radiology and the Zuckerman Mind Brain Behavior Institute, Columbia UniversityNew YorkUnited States; 4 Graduate School of Information Science and Technology, Hokkaido UniversitySapporoJapan; 5 National Institute for Physiological SciencesOkazakiJapan; 6 Exploratory Research Center on Life and Living SystemsOkazakiJapan; 7 National Institute for Basic Biology, National Institutes of Natural SciencesOkazakiJapan; 8 The Graduate School for Advanced StudyHayamaJapan; 9 Department of Biology, Faculty of Science, Kyushu UniversityFukuokaJapan; 10 RIKEN center for Advanced Intelligence ProjectTokyoJapan; Research Institute of Molecular Pathology, Vienna Biocenter and University of ViennaAustria; Emory UniversityUnited States

**Keywords:** cell tracking, bioimaging, deep learning, quantitative biology, *C. elegans*, Zebrafish

## Abstract

Despite recent improvements in microscope technologies, segmenting and tracking cells in three-dimensional time-lapse images (3D + T images) to extract their dynamic positions and activities remains a considerable bottleneck in the field. We developed a deep learning-based software pipeline, 3DeeCellTracker, by integrating multiple existing and new techniques including deep learning for tracking. With only one volume of training data, one initial correction, and a few parameter changes, 3DeeCellTracker successfully segmented and tracked ~100 cells in both semi-immobilized and ‘straightened’ freely moving worm's brain, in a naturally beating zebrafish heart, and ~1000 cells in a 3D cultured tumor spheroid. While these datasets were imaged with highly divergent optical systems, our method tracked 90–100% of the cells in most cases, which is comparable or superior to previous results. These results suggest that 3DeeCellTracker could pave the way for revealing dynamic cell activities in image datasets that have been difficult to analyze.

## eLife digest

Microscopes have been used to decrypt the tiny details of life since the 17th century. Now, the advent of 3D microscopy allows scientists to build up detailed pictures of living cells and tissues. In that effort, automation is becoming increasingly important so that scientists can analyze the resulting images and understand how bodies grow, heal and respond to changes such as drug therapies.

In particular, algorithms can help to spot cells in the picture (called cell segmentation), and then to follow these cells over time across multiple images (known as cell tracking). However, performing these analyses on 3D images over a given period has been quite challenging. In addition, the algorithms that have already been created are often not user-friendly, and they can only be applied to a specific dataset gathered through a particular scientific method.

As a response, Wen et al. developed a new program called 3DeeCellTracker, which runs on a desktop computer and uses a type of artificial intelligence known as deep learning to produce consistent results. Crucially, 3DeeCellTracker can be used to analyze various types of images taken using different types of cutting-edge microscope systems. And indeed, the algorithm was then harnessed to track the activity of nerve cells in moving microscopic worms, of beating heart cells in a young small fish, and of cancer cells grown in the lab. This versatile tool can now be used across biology, medical research and drug development to help monitor cell activities.

## Introduction

Imaging cells to reveal the dynamic activities of life has become considerably more feasible because of the remarkable developments in microscope hardware in recent years (Frigault et al., 2009; Ahrens and Engert, 2015; Bouchard et al., 2015; Weisenburger and Vaziri, 2018). In addition, multiple software platforms for processing 2D/3D still images and 2D + T images have been developed (Eliceiri et al., 2012).

However, processing cells in 3D + T images has remained difficult, especially when cells cannot be clearly separated and/or their movements are relatively large, such as cells in deforming organs. For processing objects in 3D + T images, the following two steps are required: (1) segmentation: segmenting the regions of interest in each 3D image into individual objects (Figure 1—figure supplement 1, left) and (2) tracking: linking an object in a particular volume to the same object in the temporally adjacent volume (Figure 1—figure supplement 1, right). For segmenting and tracking (hereafter collectively called ‘tracking’) cells of deforming organs in 3D + T images, programs optimized for processing images in particular conditions have been developed (Schrödel et al., 2013; Toyoshima et al., 2016; Venkatachalam et al., 2016; Nguyen et al., 2017). However, these methods cannot be used under conditions other than those for which they were designed, at least without a loss in processing efficiency. In other words, 3D + T images, especially those obtained under challenging conditions, can be efficiently processed only when customized software is developed specifically for those images. One reason for this is that many parameters must be optimized to achieve good results—For example, even in 2D image processing, changes in lighting could require the re-optimization of parameters for segmentation and tracking (Egnor and Branson, 2016).

One way to solve this problem is to optimize the parameters automatically using machine learning, especially deep learning. Deep learning methods use an artificial neural network with multiple layers, that is, a deep network, to process complex data and automatically optimize a number of parameters from training data, which allows users to easily apply a single method to images obtained under different conditions. In addition to this flexibility, deep learning methods have outperformed conventional methods in some image processing tasks such as image classification (LeCun et al., 2015; Krizhevsky et al., 2012). Nevertheless, deep learning methods are used mostly for segmentation and/or object detection, but not for tracking because of the difficulty in preparing training data (Moen et al., 2019): Correctly tracking a number of objects manually to obtain training data, especially from 3D + T images recorded over long period of time, is extremely challenging. In addition, designing a multiple-step pipeline for segmenting cells and tracking their positions to work under various conditions has been difficult.

In this study, we developed 3DeeCellTracker, a new pipeline utilizing deep learning techniques in segmentation and, for the first time to our knowledge, in tracking of cells in 3D + T images. We solved the problem of training data preparation for tracking by creating a synthetic dataset (see below). We also designed a multiple-step pipeline to achieve accurate tracking. 3DeeCellTracker was implemented on a desktop PC to efficiently and flexibly track cells over hundreds of volumes of 3D + T images recorded under highly divergent optical or imaging conditions. Using only one volume for training and one initial correction, 3DeeCellTracker efficiently tracked 100–150 neurons in the brains of semi-immobilized or ‘straightened’ (special pre-processing step based on the worm's posture) freely moving *Caenorhabditis elegans* roundworms from four image datasets, obtained using spinning disk confocal systems in three different laboratories. With a few modifications, 3DeeCellTracker also tracked ~100 cells in the naturally beating heart of a zebrafish larva monitored using swept confocally aligned planar excitation (SCAPE) microscopy, a novel oblique light sheet microscope system for very rapid 3D + T imaging (Bouchard et al., 2015; Voleti et al., 2019). Furthermore, 3DeeCellTracker tracked ~900 cells in a cultured 3D tumor spheroid, which were imaged with a two photon microscope system. Our pipeline provided robust tracking results from the above-mentioned real datasets as well as from degenerated datasets, which differed in terms of signal-to-noise ratios, cell movements, and resolutions along the *z*-axis. Notably, 3DeeCellTracker's performance was better in terms of the tracking results than those from recently developed 2D/3D tracking software running on a desktop PC, and comparable to software running on ﻿a high-performance computing cluster (Toyoshima et al., 2016; Nguyen et al., 2017; Bannon et al., 2018). Furthermore, by using the positional information of the tracked cells, we extracted dynamics of the cells: the worm's neurons exhibited complex activity patterns in the brain, the zebrafish heart cells exhibited activities synchronized with heart chamber movement, and the tumor spheroid cells exhibited spontaneous activities without stimulation. These results indicate that, 3DeeCellTracker is a robust and flexible tool for tracking cell movements in 3D + T images, and can potentially enable the analysis of cellular dynamics that were previously difficult to investigate.

## Results

### Overview

**Figure 1.**
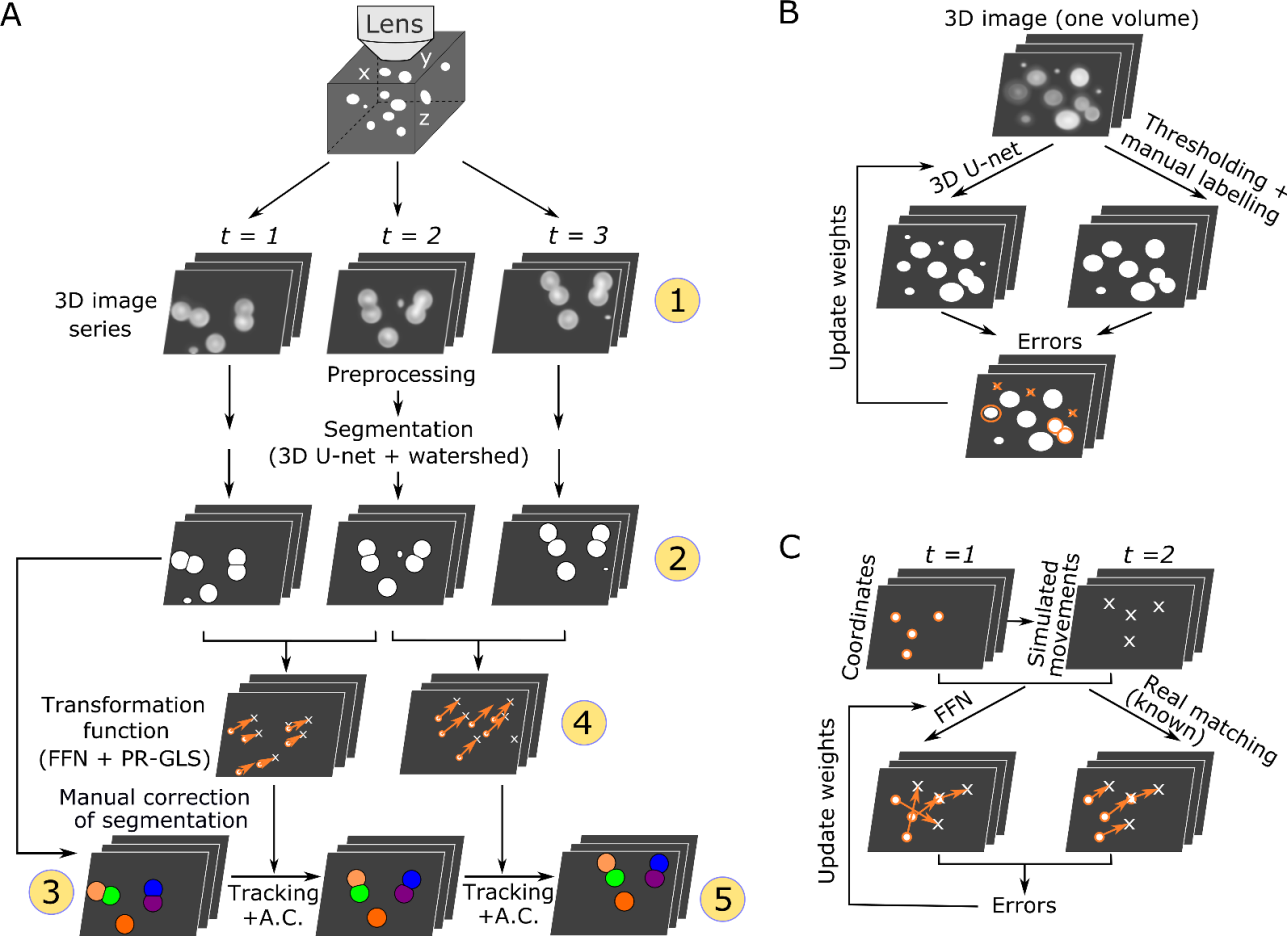
Overall procedures of our tracking method and the training procedures of the deep networks. (**A**) Procedures of segmentation and tracking. 3D + T images are taken as a series of 2D images at different *z* levels (step 1) and are preprocessed and segmented into discrete cell regions (step 2). The first volume (*t* = 1) of the segmentation is manually corrected (step 3). In the following volumes (*t* ≥ 2), by applying the inferred transformation functions generated by FFN + PR-GLS (step 4), the positions of the manually confirmed cells are successively updated by tracking followed by accurate corrections (A.C.) (step 5). The circled numbers indicate the five different procedure steps. (**B**) Procedures for training the 3D U-net. The cell-like regions predicted by 3D U-net are compared with manually labeled cell regions. The errors (orange) are used to update U-net weights. (**C**) Procedures for training the feedforward network. The movements of cells used for training are generated from simulations, thus their correct matches are known. The errors in the predictions are used to update the feedforward network weights.

We developed a new pipeline, 3DeeCellTracker, which integrates novel and existing techniques (Figure 1A). After preprocessing (see Materials and methods), it performs automatic segmentation of cells in all 3D + T images using 3D U-Net for classifying individual voxels into cell or non-cell categories (Ronneberger et al., 2015; Çiçek et al., 2016). Continuous regions of ‘cell’ voxels are separated into individual cell regions using the watershed method (Beucher and Meyer, 1993), and then numbered. The segmented cells are manually corrected only in the first volume of 3D images. In the following 3D tracking step, we considerably increased the efficiency by introducing a deep learning technique, feedforward network (FFN), to predict cell positions based on spatial patterns of cells maintained between previous and current images. The predicted positions are corrected with a non-rigid point set registration method called PR-GLS (Ma et al., 2016) and by our custom method to obtain precise cell locations, which are critical for extracting the accurate dynamics of cellular signals. The 3D U-Net and the FFN are pre-trained using manually confirmed data or synthetic data (Figure 1B,C) from a single 3D image volume. The tracking results were visually inspected by comparing the locations of tracked cells with the corresponding raw images (Figure 1—figure supplement 2). The keys to our method are the use of simulation to produce large amounts of training data for the FFN and the carefully designed post-processing methods for the FFN, which result in the flexible and robust tracking of moving cells in very different 3D + T datasets. In the following two sections, we describe details of the segmentation and tracking method.

**Figure 1—figure supplement 1.**
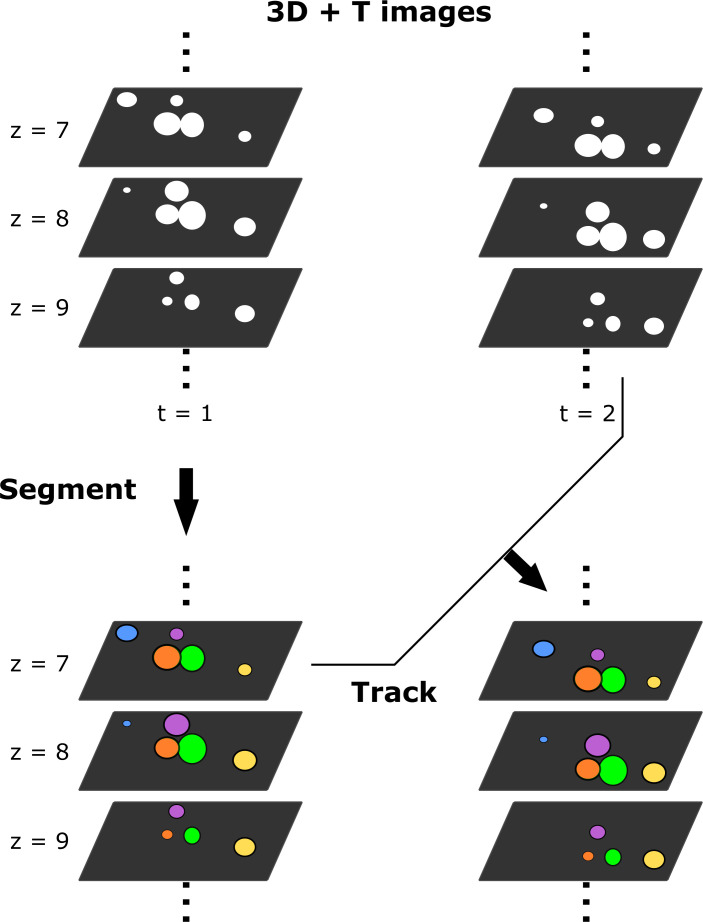
Illustration of segmentation and tracking. Cell nuclei labeled with a fluorescent protein are scanned into 3D + T images and segmented into individual nuclei. The positions of segmented nuclei varied over time due to organ movement and/or deformation. These nuclei are tracked by updating their positions.

**Figure 1—figure supplement 2.**
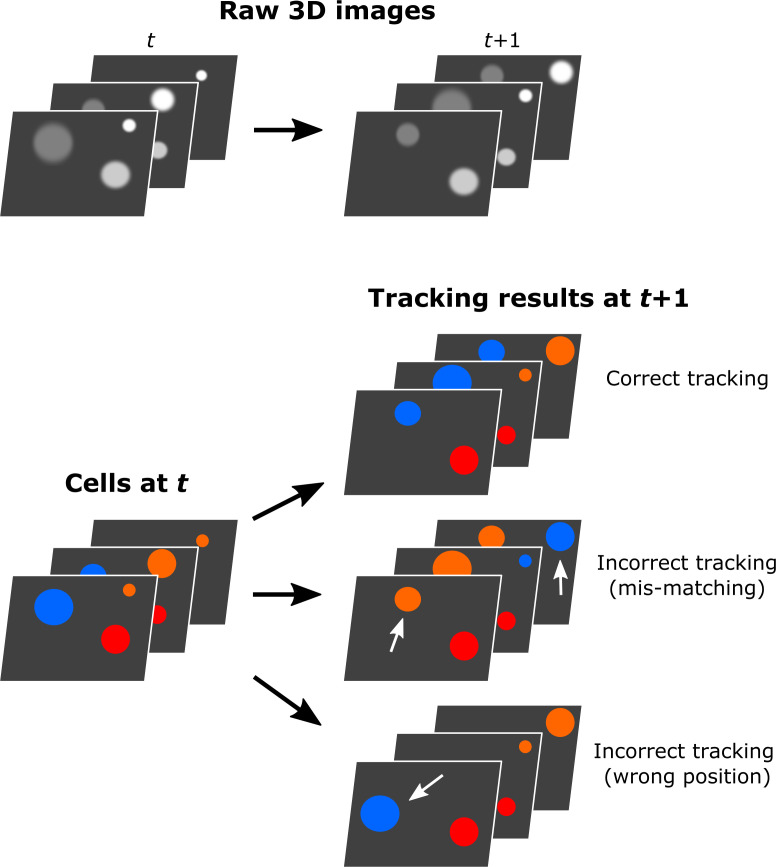
Illustration of the visual inspection of the tracking results. The tracking results were compared with the raw images in each volume visually to find tracking mistakes.

### Segmentation

**Figure 2.**
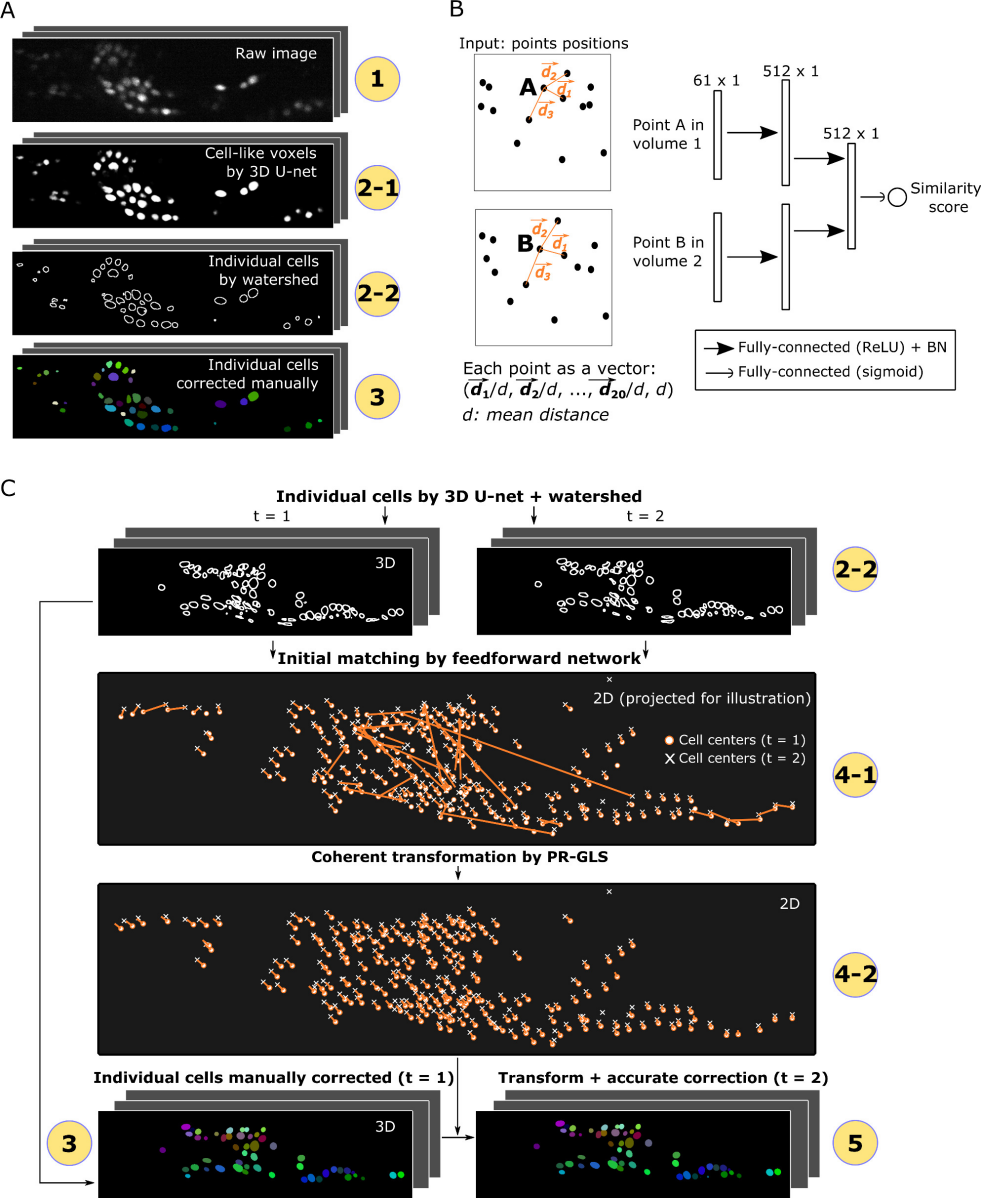
Detailed descriptions of segmentation and tracking. (**A**) Details of segmentation. The circled numbers correspond to the numbers in Figure 1, while the numbers 2–1 and 2–2 indicate cell-like voxels detected by 3D U-net and individual cells segmented by watershed, respectively. One volume of a worm neuron dataset is used as an example. (**B**) Left: Definition of the positions in two point sets corresponding to two volumes. Right: Structure of the feedforward network for calculating the similarity score between two points in two volumes. The numbers on each layer indicate the shape of the layer. ReLU: Rectified linear unit. BN: Batch normalization. (**C**) Details of tracking. 4–1 and 4–2 indicate the initial matching from *t* = 1 to *t* = 2 using our custom feedforward network and the more coherent transformation function inferred by PR-GLS, respectively. The orange lines in 4–1 and 4–2 indicate the inferred matching/transformation from *t* = 1 to *t* = 2.

For segmentation, cell-like regions in an image should be segmented into individual cells which may differ in intensities, sizes, shapes, and textures. Cell segmentation in 2D images using deep networks has been previously reported (Ronneberger et al., 2015; Van Valen et al., 2016; Bannon et al., 2018). In this study, we utilized a deep network called 3D U-Net to segment cells in 3D images to predict the class labels (cell or non-cell) of individual voxels based on information contained in neighboring voxels (Figure 2A and Figure 2—figure supplement 1; Ronneberger et al., 2015; Çiçek et al., 2016). The U-Net can generate precise segmentation under diverse imaging conditions and can be trained with very few annotated images (e.g. only one 3D image volume in this study; see Figure 1B).

**Figure 2—figure supplement 1.**
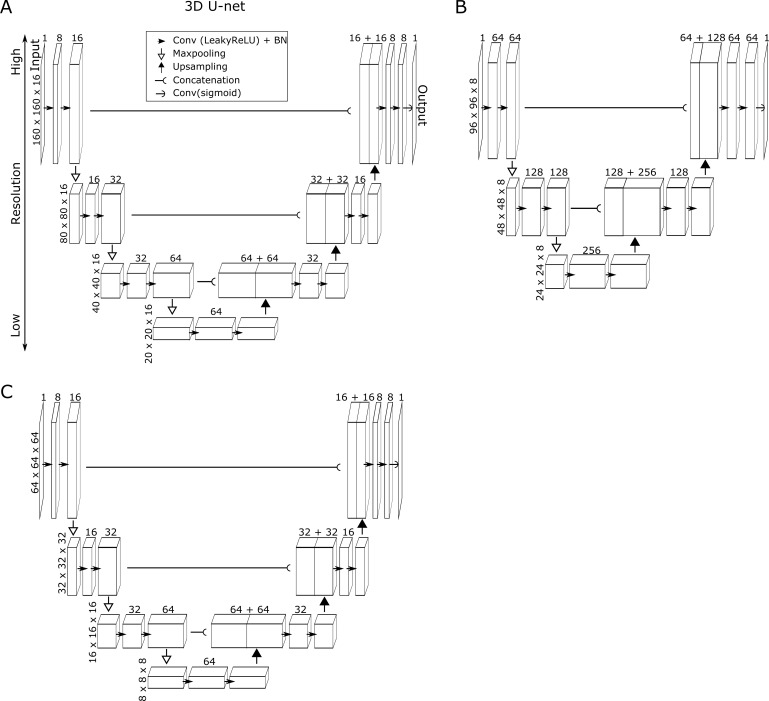
Structures of the 3D U-Net used in this study. We designed different structures for adopting different resolutions of images, which is preferred but not necessary. End-users can freely choose one of the structures for their own data for simplicity. (**A**) Used for datasets worm #1 and #2, and for the binned zebrafish dataset. (**B**) Used for dataset worm #3 and the degenerated worm datasets. (**C**) Used for the raw zebrafish dataset. Each raw 3D image was split into sub-images and then sent into the network as input (top left) to generate maps of cell regions as output (top right), finally these maps for sub-images were combined. In the network, images were filtered and compressed to lower-resolution then recovered to the original resolution again, in order to utilize both local and global features. Numbers on each intermediate layer indicate the number of convolutional filters, while numbers on the left of each row indicate the size of the 3D images of input, output, and intermediate layers. Conv: Convolution; BN: Batch normalization.

**Figure 2—figure supplement 2.**
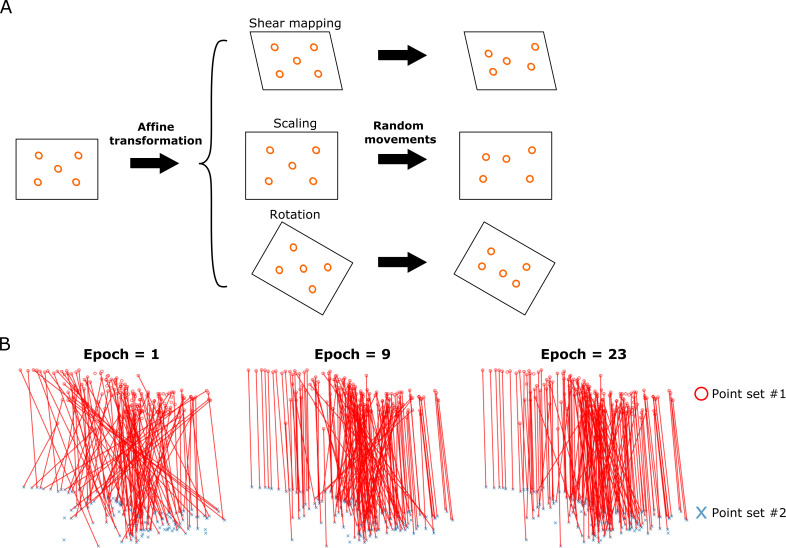
Illustration of the FFN training method. (**A**) Generation of the synthetic data for training the FFN. The locations of a point set (orange circles) were transformed by affine function with different parameters, and additional movements of the transformed points were incorporated by adding random movements to each point. (**B**) The matching by FFN between two test point sets, which was improved in accuracy over the course of training.

**Figure 2—figure supplement 3.**
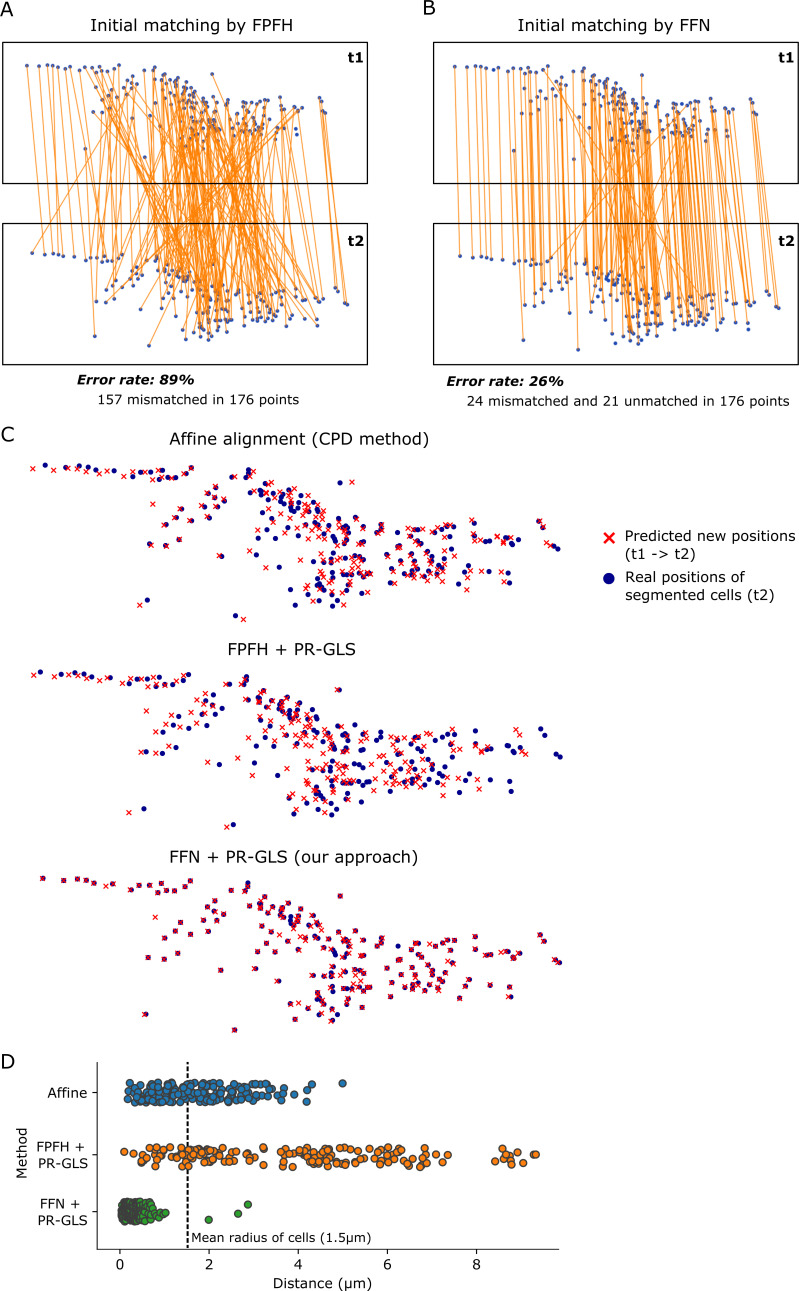
Comparison of our method with previous tracking techniques. (**A and B**) Comparison of the initial matchings made by FPFH (Rusu et al., 2009) and FNN. Cell centers were generated from the 3D U-net + watershed segmentation of two volumes in worm #3 dataset. Blue dots indicate cell centers, and orange lines indicate the estimated matches between these two point sets. We manually counted the error rates. In the regular process, matching is between temporally adjacent volumes. In this panel, we performed matching between the points separated for 10 volumes (t1 and t2) as an example of a challenging condition. (**C**) The predicted positions from the three methods (affine alignment, FPFH + PR-GLS, and FFN + PR-GLS) and the real positions of cells at *t2*. (**D**) The distances between the predicted positions and their real positions for the three methods.

**Figure 2—figure supplement 4.**
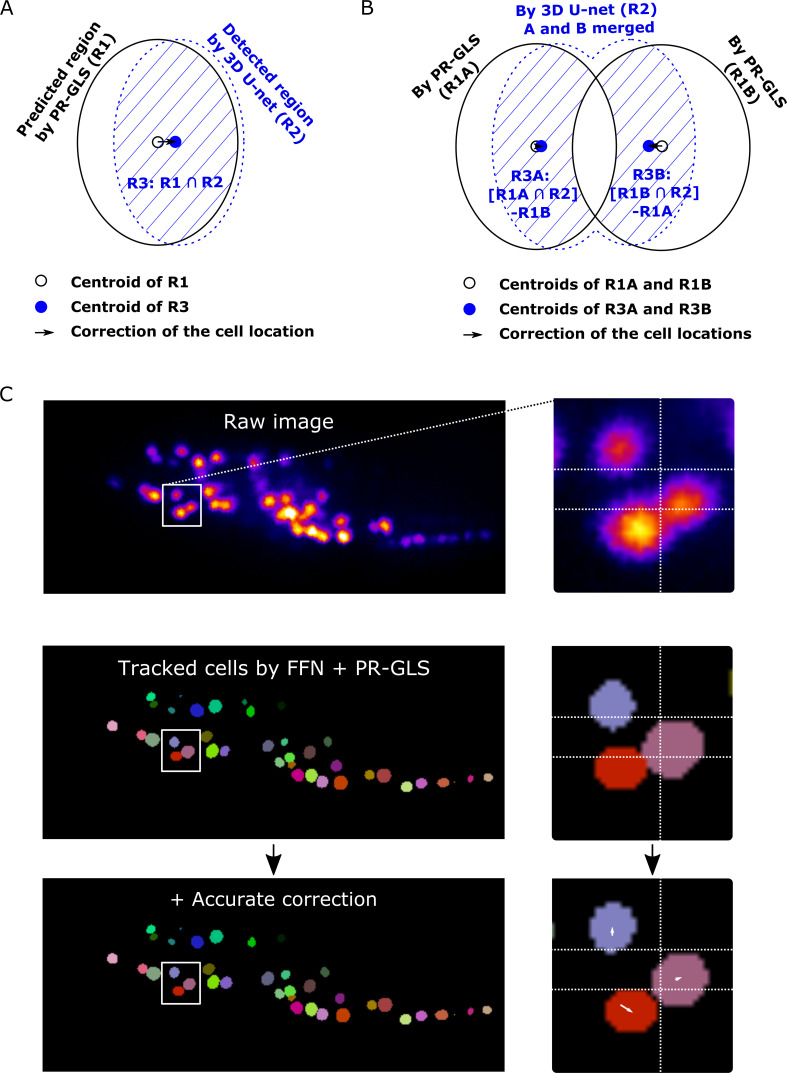
Illustration of the accurate correction. (**A and B**) Our method for accurately correcting cell locations. (**A**) Correction method in the single cell case. We obtained the region (the large solid black ellipse) and the center (small black circle) of a cell predicted by PR-GLS from the previous volume. We also obtained cell regions detected by 3D U-Net (the dashed blue ellipse). Subsequently, we calculated the intersection of the PR-GLS region and 3D U-Net regions (filled with diagonal blue lines) and its center (small blue circle). The final accurate correction follows the small black arrow, that is the center of the cell should move from the small black circle to the small blue circle. (**B**) Correction method when two regions predicted by PR-GLS partially overlapped. The method is essentially the same as in (**A**), except that areas where two PR-GLS regions overlapped were removed from the intersection region of PR-GLS and 3D U-net, generating two smaller regions (diagonal blue lines). We used this technique to prevent the two cells from merging in tracking results. (**C**) An example of accurate correction with a worm neuron dataset. The locations of the cells predicted by FFN + PR-GLS (middle) had small discrepancies with their real positions in the raw image (top). Such discrepancies were corrected (white arrows) by our method (bottom). All panels are correspoinding to a 2D layer of a 3D image at *z* = 66.

The pre-processed images of the first volume are used to train the 3D U-Net (Figure 1B), and the trained U-net is then used for the segmentation of cells in all the following volumes. Once trained on one dataset, the 3D U-Net can be directly reused for different datasets obtained under similar optical conditions. The cell-like regions detected using 3D U-Net are grouped and separated into individual cells using the watershed method (see Figure 2A and Materials and methods).

### Tracking

For tracking cells, two major strategies can be considered. One strategy is to utilize the information contained in each cell region, for example, local peaks or distributions of intensities (Toyoshima et al., 2016). Using this information, a program can update the position of each cell by searching for its new position in nearby regions in the next volume. However, the obvious drawback of this strategy is that cells can be mistracked if their movements are comparable to or greater than the distances between adjacent cells (see below).

Another strategy is to represent cells by their center points, ignoring the information in each cell region and treating the cells as a set of points. In this strategy, the new positions of a cell set in the next volume can be estimated based on the patterns of their spatial distributions, and cells with large movements can also be tracked based on the global trend of the movements. Previously reported methods utilizing this strategy, called point set registration (Ma et al., 2016; Jian and Vemuri, 2005; Myronenko et al., 2006), used the spatial distributions and coherency of movements to track points within artificial datasets. However, the spatial patterns are conventionally characterized by features designed by experts, an approach that did not work well for the cell images used in this study (refer to the results below concerning Fast Point Feature Histograms [FPFH; Rusu et al., 2009] versus FFN). Another problem with this strategy is that the estimated positions are not always accurate because the information contained in each cell region is ignored (see the results below concerning our method for accurate correction).

In order to obtain more robust and accurate tracking results, we integrated the spatial pattern (i.e. point sets) and local cell region strategies and used a deep learning technique, FFN, for the former. We used FFN to match temporally adjacent cells based on the distance pattern between each cell and its 20 surrounding cells (Figure 2B). Using the pattern obtained by the FFN, all the cells at *t*_1_ are compared with all the cells at *t*_2_, and the most similar ones are regarded as the same cells at *t*_1_ and *t*_2_, a process we call initial matching.

Although deep network techniques are expected to produce superior cell tracking results, the method had not been used previously because it requires large amounts of training data. These data are difficult to prepare manually, especially for 3D + T images, as validating and correcting large numbers of cell positions over time by switching multiple layers along the *z* axis is virtually impossible. To solve this difficulty in preparing training data for FFN, we generated >500,000,000 synthetic training data points by simulating cell movements (see Figure 2—figure supplement 2 and Materials and methods).

In the pipeline, the center points of cells are extracted from the cell regions segmented by 3D U-Net and the watershed method, and a pre-trained FFN (Figures 1C and 2B) is applied to the cell points to generate the initial matching from volumes *t* to *t*+1 (Figure 2C, panels 2–2 and 4–1). To improve the initial matching, a non-rigid point set registration method (PR-GLS) (Ma et al., 2016) is used to generate a coherent transformation, that is, neighboring cells should have similar movements (Figure 2C, panel 4–2). Originally, PR-GLS was used in conjunction with the FPFH method to predict cell positions (Ma et al., 2016); however, our FFN generates more accurate initial matchings than FPFH (Figure 2—figure supplement 3A and B), and our combination of FFN + PR-GLS generates more accurate predictions of cell positions than the FPFH + PR-GLS or the classic affine alignment (Myronenko et al., 2006) does (Figure 2—figure supplement 3C and D). Nevertheless, our method sometimes generates subtle errors because some cells may show slightly different movements from those of their neighboring cells, which can accumulate over time. To overcome these errors, the estimated positions are accurately corrected to compensate for their differences by utilizing information from local cell regions contained in the 3D U-Net output (Figure 2C, panel 5, Figure 2—figure supplement 4, and Materials and methods).

### Tracking neurons in the deforming worm’s whole brain

To test the performance of 3DeeCellTracker, we analyzed 3D + T images of neurons in the deforming brain of *C. elegans. C. elegans* has been used as a model for imaging all neuronal activities in the brain (‘whole brain imaging’) owing to its small brain (~40 µm^3^ in an adult) in a transparent body, the complete description of all connections of 302 neurons, feasibility in the use of genetically encoded calcium indicators (GECI), and the capability of perception, memory, and decision-making in its small brain (de Bono and Maricq, 2005). Whole brain imaging of *C. elegans* has been reported from several laboratories, and the most popular optical system currently is the spinning disk confocal system, for which each laboratory has developed their own tracking software (Schrödel et al., 2013; Toyoshima et al., 2016; Venkatachalam et al., 2016; Nguyen et al., 2017). We established our own spinning disk confocal system for whole-brain imaging, OSB-3D (see Materials and methods for details), and obtained 3D + T images of whole brain activity from a strain established in our laboratory and from the one used in a previous study by Nguyen et al., 2016 (datasets worm #1 and #2, respectively). In addition, we analyzed the whole-brain images published previously using a different optical system (Toyoshima et al., 2016; dataset worm #3). In all datasets, the worms were semi-immobilized either by anesthetization (worm #1 and #2) or by constriction (worm #3), and red fluorescent proteins and GECI were used to monitor cell positions and cell activities, respectively.

**Figure 3.**
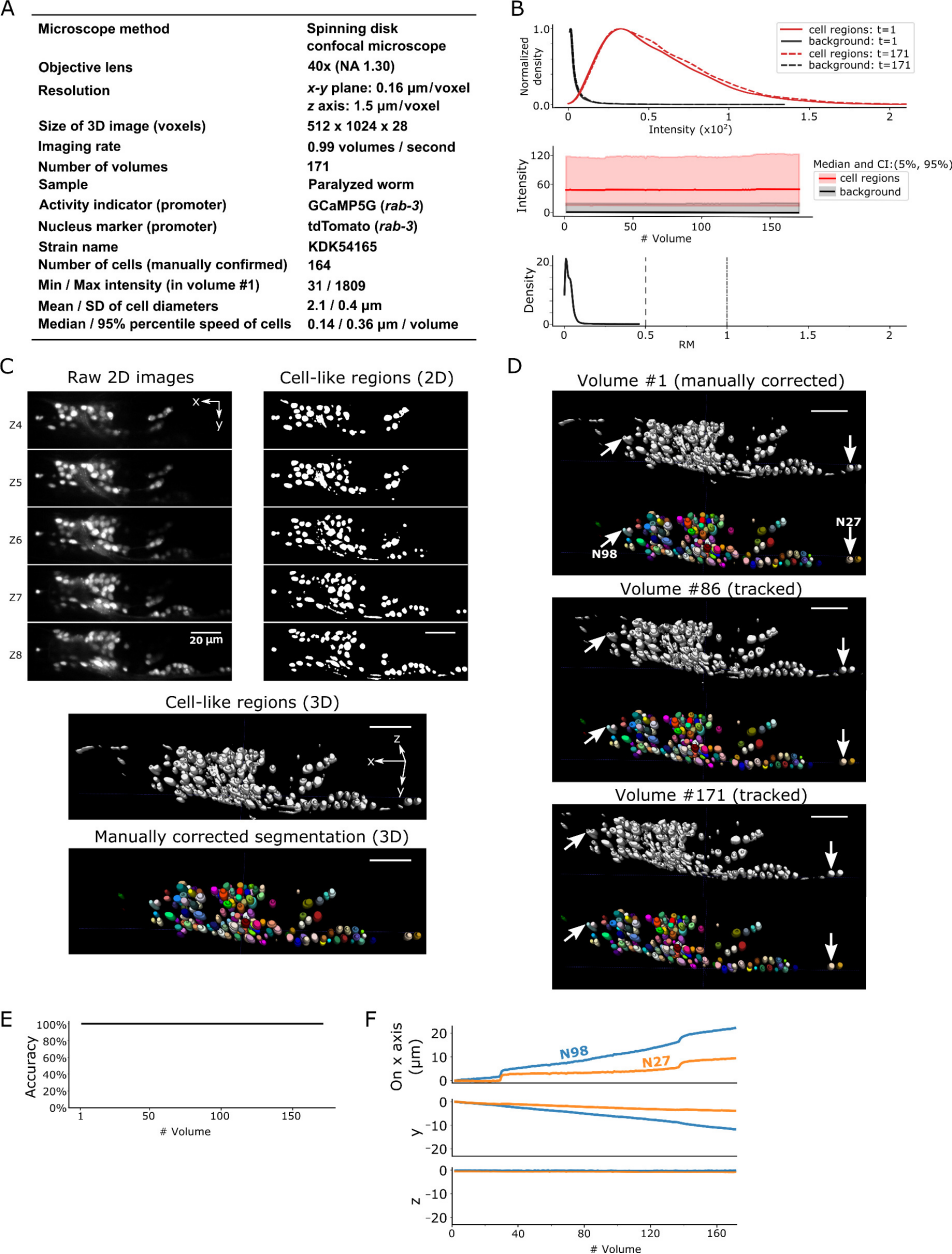
Results for dataset worm #1a. (**A**) Experimental conditions. (**B**) The distribution (top) and the time-course changes (middle) of the intensities in cell regions and in the background, and the distribution of relative movements (bottom). The distributions were estimated using the ‘KernelDensity’ function in Scikit-learn library in Python. The intensities were calculated using 8-bit images transformed from the 16-bit raw images. The relative movement (*RM*) is the normalized cell movement: *RM* = movement/(distance to the closest cell). The critical values, *RM* = 0.5 and 1.0, are emphasized by dashed and dash-dotted lines, respectively (see Figure 4 and Materials and methods for details). CI: confidence interval. (**C**) 3D image and its segmentation result in volume #1. Top left: Five layers (Z4–Z8) of the raw 2D images. Top right: Cell-like regions corresponding to the 2D images at left, detected by the 3D U-Net. Middle: Cell-like regions of the 3D view including all layers. Bottom: Final segmentations using watershed plus manual correction. Different colors represent different cells. (**D**) Tracking results. Tracked cells in volume #86 and volume #171 are shown, which are transformed from the segmentation in volume #1. In each panel, the top shows the cell-like regions detected by 3D U-Net, and the bottom shows tracked cells. Arrows indicate two example cells with large (N98) and small movement (N27). All cells were correctly tracked. (**E**) The tracking accuracy through time. (**F**) Movements of the two representative cells N98 and N27 in *x*, *y*, and *z* axes, indicating an expansion of the worm mainly in the *x-y* plane. The initial positions at volume #1 were set to zero for comparison. All scale bars, 20 µm.

After segmentation, we manually confirmed 164 neurons in the head in the first volume of 3D + T images, in which the distribution of cell signals was mostly, but not completely, separated from the background signals (dataset worm #1a; see Figure 3A–C). While the worm was anesthetized, its head deformed and the cells moved during imaging. Our method tracked all the neurons in all 171 volumes except for those that moved out of the field of view of the camera (Figure 3D–F, Figure 3—video 1).

**Figure 3—figure supplement 1.**
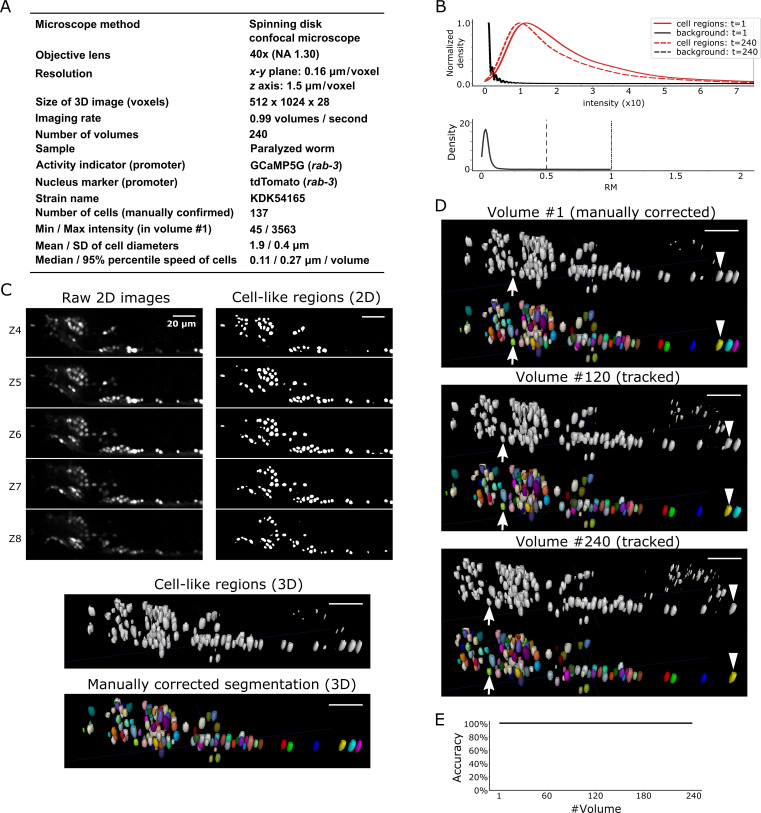
Results for dataset worm #1b. (**A**) Experimental conditions. (**B**) Intensities and movements. (**C**) 3D image and its segmentation result in volume #1. (**D**) Tracking results. Tracked cells in volume #120 and volume #240 are shown. Arrows and arrowheads indicate two tracked cells. All the cells were correctly tracked except for those that moved out of the field of view of the camera. (**E**) Tracking accuracy through time. All scale bars, 20 µm.

**Figure 3—figure supplement 2.**
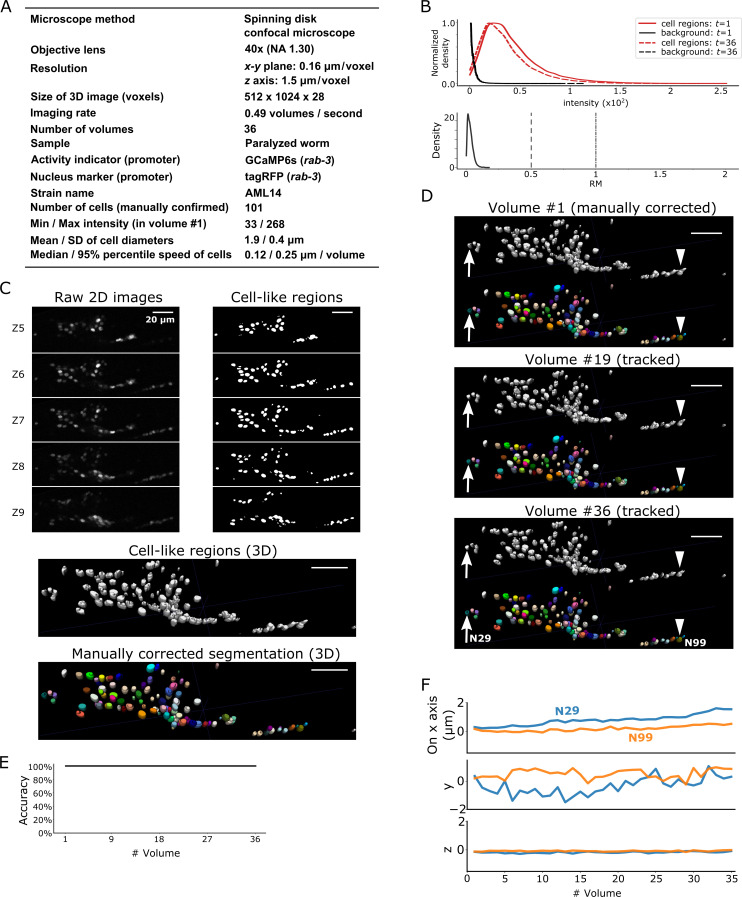
Results for dataset worm #2. (**A**) Experimental conditions. (**B**) Intensities and movements. (**C**) 3D image and its segmentation result in volume #1. (**D**) Tracking results. Tracked cells in volume #19 and #36 are shown. Arrows and arrowheads indicate two tracked cells. All cells were correctly tracked. (**E**) Tracking accuracy through time. (**F**) Movements of the two representative cells N29 and N99. When compared with Figure 3F, these cells showed small but irregular movements in the *x-y* plane. All scale bars, 20 µm.

**Figure 3—figure supplement 3.**
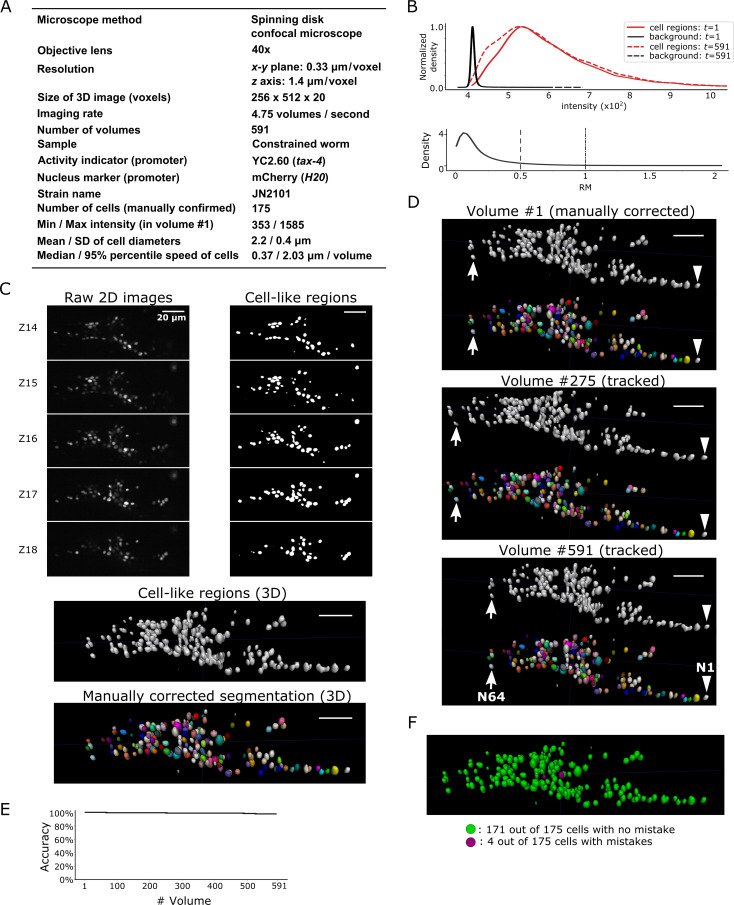
Results for dataset worm #3. (**A**) Experimental conditions. (**B**) Intensities and movements. (**C**) 3D image and its segmentation result in volume #1. (**D**) Tracking results. The tracked cells in volume #275 and #591 are shown. Arrows and arrowheads indicate two tracked cells. For their movements, see Figure 9L. Only four neurons were mistracked. (**E**) Tracking accuracy through time. (**F**) The positions of cells with/without tracking mistakes. Some small cells are invisible. All scale bars, 20 µm.

**Figure 3—video 1.** Tracking results for dataset worm #1a. The animation of cell regions (top) detected by 3D U-net and corresponding individual cells (bottom) in dataset #1a tracked by our method.

**Figure 3—video 2.** Tracking results for dataset worm #2. The animation of cell regions (top) detected by 3D U-net and corresponding individual cells (bottom) in dataset #2 tracked by our method.

**Figure 3—video 3.** Tracking results for dataset worm #3. The animation of cell regions (top) detected by 3D U-net and corresponding individual cells (bottom) in dataset #3 tracked by our method.

**Figure 4.**
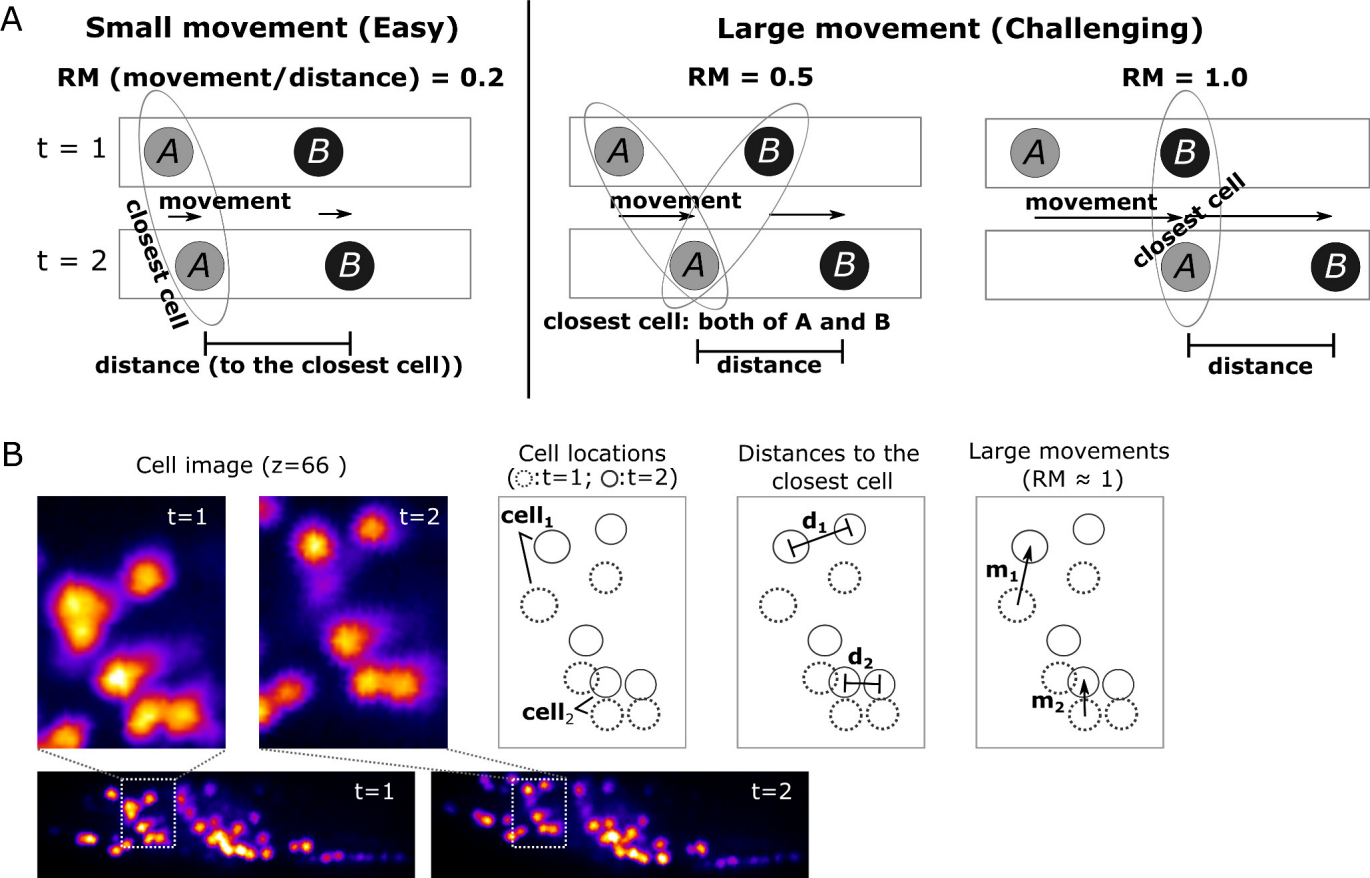
Quantitative evaluation of the challenging relative movement (*RM*) of cells. (**A**) Illustrations of movements in one dimensional space with *RM* = 0.2, 0.5, and 1.0. Here *RM* = (movement a cell traveled per volume)/(distance to the closest neighboring cell). When *RM* ≥0.5, cell A at *t* = 2 will be incorrectly assigned to cell B at *t* = 1 if we simply search for its closest cell instead of considering the spatial relationship between cells A and B. When *RM* = 0.5, cell assignment is also impossible. Please also see Materials and methods for details. (**B**) Examples of large cell movements with *RM* ≈ 1. Also see Figure 10 and Table 3.

To evaluate the difficulty in tracking cells with large movements, we calculated a score for the relative movements (*RM*), which is the movement of a cell divided by the distance from that cell to its closest neighboring cell. When *RM* is small, searching for the closest cell is the simplest method to find the new position of a given cell in the next volume. However, such a simple approach may lead to tracking errors when *RM* ≥0.5 (Figure 4 and Materials and methods). Although most of the cell *RM* values were ≤0.5 in the worm #1 dataset (Figure 3B, bottom), other datasets had cells with *RM* ≥0.5; nevertheless, these were also successfully tracked by our program (see below).

**Figure 5.**
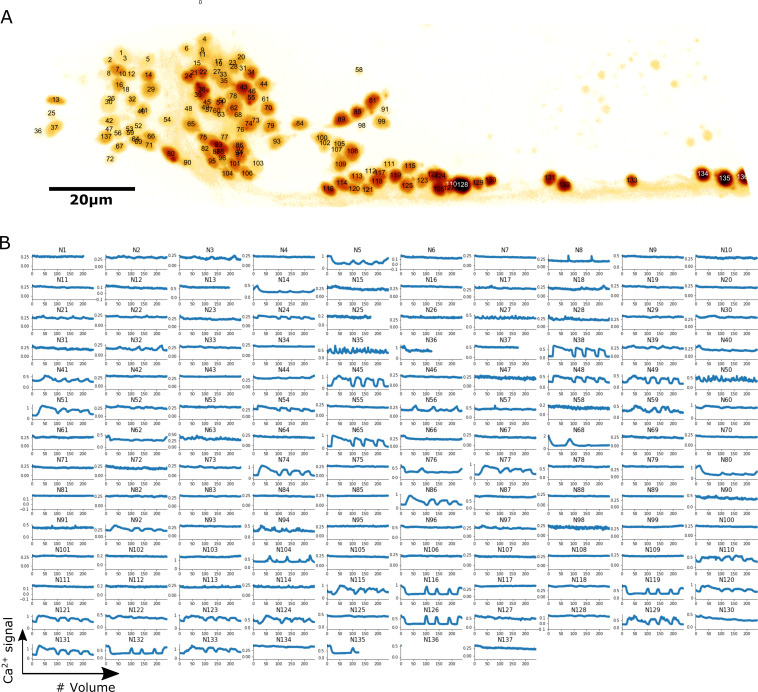
Localization and calcium dynamics in all 137 neurons for dataset worm #1b. (**A**) The ID numbers of neurons were superimposed on the image of cell nuclei (tdTomato) projected onto the 2D plane. The orientation of the image has been rotated to align the anterior-posterior axis with the horizontal direction. To increase the visibility of both nuclei and IDs, we inverted the intensity of the nuclei image and displayed it using the pseudocolor ‘glow’. (**B**) Extracted calcium dynamics (GCaMP / tdTomato) in all 137 neurons. All cells were correctly tracked, while some of them moved out of the field of the camera and thus their activities were recorded for shorter periods.

We also tested worm #1b dataset, obtained from the same strain with worm #1a (Figure 3—figure supplement 1), in which we again achieved 100% tracking accuracy without changing any parameters aside from the noise level (see Tables 1 and 2). The positional information of cell nuclei in both datasets was then used to extract the calcium dynamics of the neurons based on GECI intensities, which reflect spontaneous activities of neurons in the worm's brain (Figure 5, Figure 5—figure supplement 1 and Figure 5—video 1).

**Figure 5—figure supplement 1.**
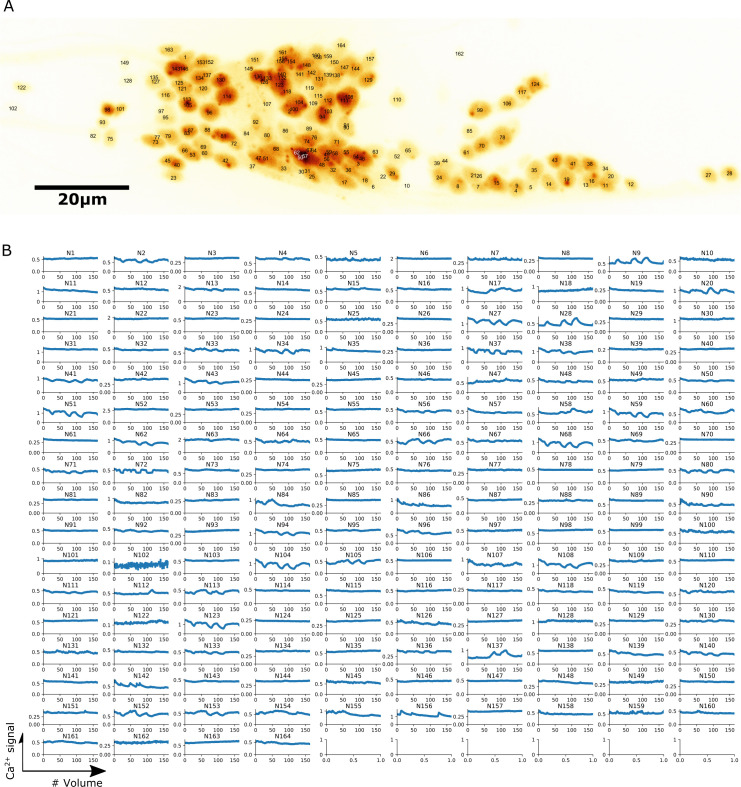
Localization and calcium dynamics in all 164 neurons for dataset worm #1a. (**A**) The ID numbers of neurons were superimposed on the image of cell nuclei (tdTomato) projected onto the 2D plane. (**B**) Extracted activities (GCaMP/tdTomato) in all 164 neurons.

**Figure 5—video 1.** GCaMP signals in dataset worm #1a. The dynamics and movements of GCaMP signals in dataset #1a projected onto the 2D plane. The intensity was displayed using the pseudocolor ‘fire’.

**Figure 5—video 2.** GCaMP signals in dataset worm #2. The dynamics and movements of GCaMP signals in dataset #2 projected onto the 2D plane.

**Table 1.** Comparison between the complexities of our method and a previous method. For each method, the procedures are listed along with the number of parameters (in bold) to be manually determined by researcher (see the guide in our GitHub repository). Our method requires manual determination of less than half of the parameters required by the previous method.

	Our method		Toyoshima et al., 2016	
	Procedures	Parameters	Procedures	Parameters
Pre-processing	Alignment	-	Alignment	-
	Local contrast normalization	**(1) Noise level**	Denoising (median filter)	**(1) Radius**
			Background subtraction	**(2) Radius**
			Gaussian blurring	**(3) Radius**
Segmentation	3D U-net (detect cell/non-cell regions)	Automatically learned	Thresholding	**(4) Method (e.g., ‘mean’,’ triangle’)**
			Watershed 1	**(5) Radius** **(6) Minimum size of cells**
	Watershed (twice)	**(2) Minimum size of cells**	Counting number of negative curvature	**(7) Threshold** **(8) Distance to borders**
			Watershed 2	**-**
Tracking	Feedforward network (generate an initial matching)	Automatically learned	Least squares fitting of Gaussian mixture	**(9-11) Default value of covariance matrix (3d vector)**
	PR-GLS (generate a coherent transformation from the initial matching)	**(3,4) Coherence level controlled by β and λ** **(5) Maximum iteration**		
	Accurate correction using intensity information	-	Removing over-segmentation	**(12) Distance**

**Table 2.** Values of parameters used for tracking each dataset. Noise levels are more variable and dependent on image quality and the pre-trained 3D U-net. Other parameters are less variable and dependent on the sizes of cells or the coherence levels of cell movements, thus do not need to be intensively explored for optimization when imaging conditions are fixed. See the user-guide in GitHub for how to set these parameters. 3D U-net structure corresponds to Figure 2—figure supplement 1A,B and C.

Dataset		Resolution (µm/voxel)	3D U-net structure	Parameters				
				Noise level	Minimum size of cells	Coherence level: β	Coherence level: λ	Maximum iteration
Worm #1a		*x-y*: 0.16 *z*:1.5	A	20	100	300	0.1	20
Worm #1b				8				
Worm #2				20				
Worm #3		*x-y*: 0.33 *z*: 1.4	B	70	10	150		20
Worm #3 + noise:	sd = 60			190				
	sd = 100			320				
	sd = 140			450				
Worm #3 + reduced sampling	1/2			70				
	1/3							
	1/5							
Worm #4 (freely moving)		~0.3 (estimated)	B	200	400	1000	0.00001	50
Zebrafish	raw	*x*: 0.86 *y*: 1.07 *z*: 2.09	C	200	40	50	0.1	50
	binned	*x*: 0.86 *y*: 1.07 *z*: 4.18	A	1	10			
Tumor spheroid		*x-y*: 0.76 *z*: 4.0	B	500	100	1000	0.00001	50

The same method was applied to 3D + T images obtained using the same OSB-3D system in our laboratory but from the neurons of a worm strain used in a previous whole brain imaging study (Nguyen et al., 2016; AML14 strain, dataset worm #2; Figure 3—figure supplement 2A). Our method again achieved a 100% accuracy of tracking and extracted calcium signals from all 101 neurons even though this dataset differs considerably from the worm #1 dataset in terms of nuclear marker intensity and movement patterns (Figure 3—figure supplement 2A–F, Figure 3—video 2 and Figure 5—video 2). It should be noted that more neurons were detected using our method than using the original method (approximately 90 neurons or less) (Nguyen et al., 2016).

We also applied our method to a publicly available dataset, which was obtained using a different strain, a different microscopy setup, and different imaging conditions from worm #1 and #2 datasets (worm #3, Figure 3—figure supplement 3A; Toyoshima et al., 2016). In this case, the worm was loosely constrained in a microfluidic device and not anesthetized, thus exhibited larger head deformations and cell displacements between volumes (by a factor of approximately three times) compared to the worm #1 and #2 datasets (Figure 3—figure supplement 3A and B). In addition, this dataset had a lower resolution (half the resolution in the *x* and *y* directions). Nevertheless, with a few parameter modifications (Table 2 and Materials and methods), our method correctly tracked 171/175 (=98%) of the neurons (Figure 3—figure supplement 3C–F and Figure 3—video 3). Our result was comparable to that of the original report, in which 171 out of 198 neurons were tracked without error (Toyoshima et al., 2016). These results indicate that our method can flexibly process 3D + T images of neurons in a semi-immobilized worm's brain obtained under different conditions.

### Tracking neurons in freely moving worm’s brains

To reveal the relationships between neuronal activities and animal behavior, Nguyen et al., developed a method to track neurons in a freely moving worm, in which the deformation of the brain and the movements of neurons are considerably larger than those occurring in semi-immobilized worms (Nguyen et al., 2016; Nguyen et al., 2017). After ‘straightening’ the worm and segmentation of its neurons, they made a set of reference volumes to which each neuron was matched to assign its identity. Although this method is powerful, it requires a high-performance scientific computing cluster running on up to 200 cores simultaneously. We therefore tested whether the 3DeeCellTracker implemented on a desktop PC could process such a challenging dataset (worm #4).

**Figure 6.**
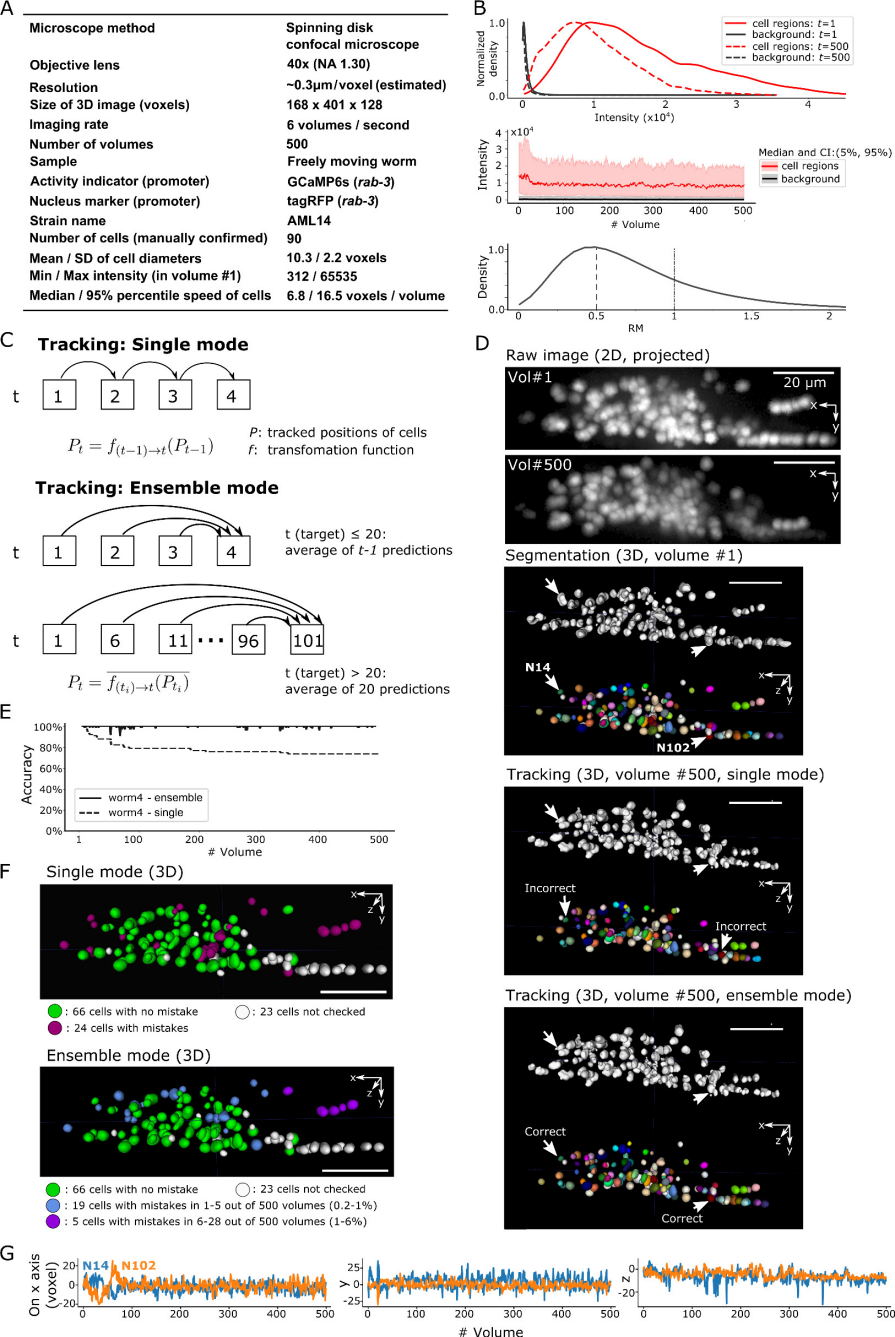
Results for dataset worm #4 – ‘straightened’ freely moving worm. (**A**) Experimental conditions. (**B**) Intensities and movements. (**C**) The two methods (single mode and ensemble mode) we used for tracking this dataset. (**D**) The results of segmentation and tracking using the two methods. The intensity of the raw images (top) was enhanced for the visibility (adjusted ‘gamma’ value from 1 to 0.5). (**E**) The tracking accuracy through time. (**F**) The positions of 90 cells with/without tracking mistakes in the single (top) and ensemble (bottom) modes. (**G**) Movements of the two representative cells N14 and N102 in *x*, *y*, and *z* axes. All scale bars, 20 µm.

With a few modifications (see Materials and methods), we used 3DeeCellTracker to segment and track 113 cells in the initial 500 volumes from the worm #4 dataset. Here we analyzed the images preprocessed by the straightening method (Nguyen et al., 2017), which is necessary for our current method. Even after the straightening, the cell movements were quite large, with many comparable to the distances between cells, that is, *RM* ≥0.5 (Figure 6A and B). We visually inspected the tracking of 90 cells while the remaining 23 were not checked because of difficulties arising from weak intensities/photobleaching and/or the cells being in dense regions. Note that 70 cells were inspected in the original report (Nguyen et al., 2017). We found that 66 out of 90 cells (73%) were correctly tracked without errors (Figure 6D–F, single mode), which is acceptable but not ideal.

**Figure 6—video 1.** Tracking results for the ‘straightened’ freely moving dataset worm #4. The animation of cell regions (top) detected by 3D U-net and corresponding individual cells (bottom) in dataset #3 tracked by our method in ensemble mode.

To further improve our method for the larger cell movements, we developed a new mode, in which multiple predictions of cell positions are made from different previous time points, and the final prediction is taken as the average of these predictions. We call this ‘ensemble mode’ and the previous method ‘single mode’, wherein the predictions of cell positions at time *t* are derived from the results at *t*-1 (Figure 6C).

When applying the ensemble mode, we again found 66 cells correctly tracked without errors, and that the remaining 24 cells were correctly tracked in most volumes (94%–98.2%). This was a substantial improvement over the single mode, in which errors at *t* are maintained until the end of tracking (Figure 6D–F, ensemble mode; Figure 6—video 1). In total, the ensemble mode correctly tracked 44,905 out of 45,000 cell movements (99.8%), a result at least comparable to that in the original report (Nguyen et al., 2017). From the trajectories of two example cells, we found that large movements, including ones along the z-axis (across ~30 layers), occurred frequently (Figure 6G), demonstrating the excellent performance of our method on a desktop PC in this challenging dataset containing considerably large scale movements. However, because the ensemble mode requires longer times for the tracking than the single mode (10 versus 2 min/volume, respectively), we used the single mode in the following analyses.

### Tracking cells in beating zebrafish heart images obtained using the SCAPE system

**Figure 7.**
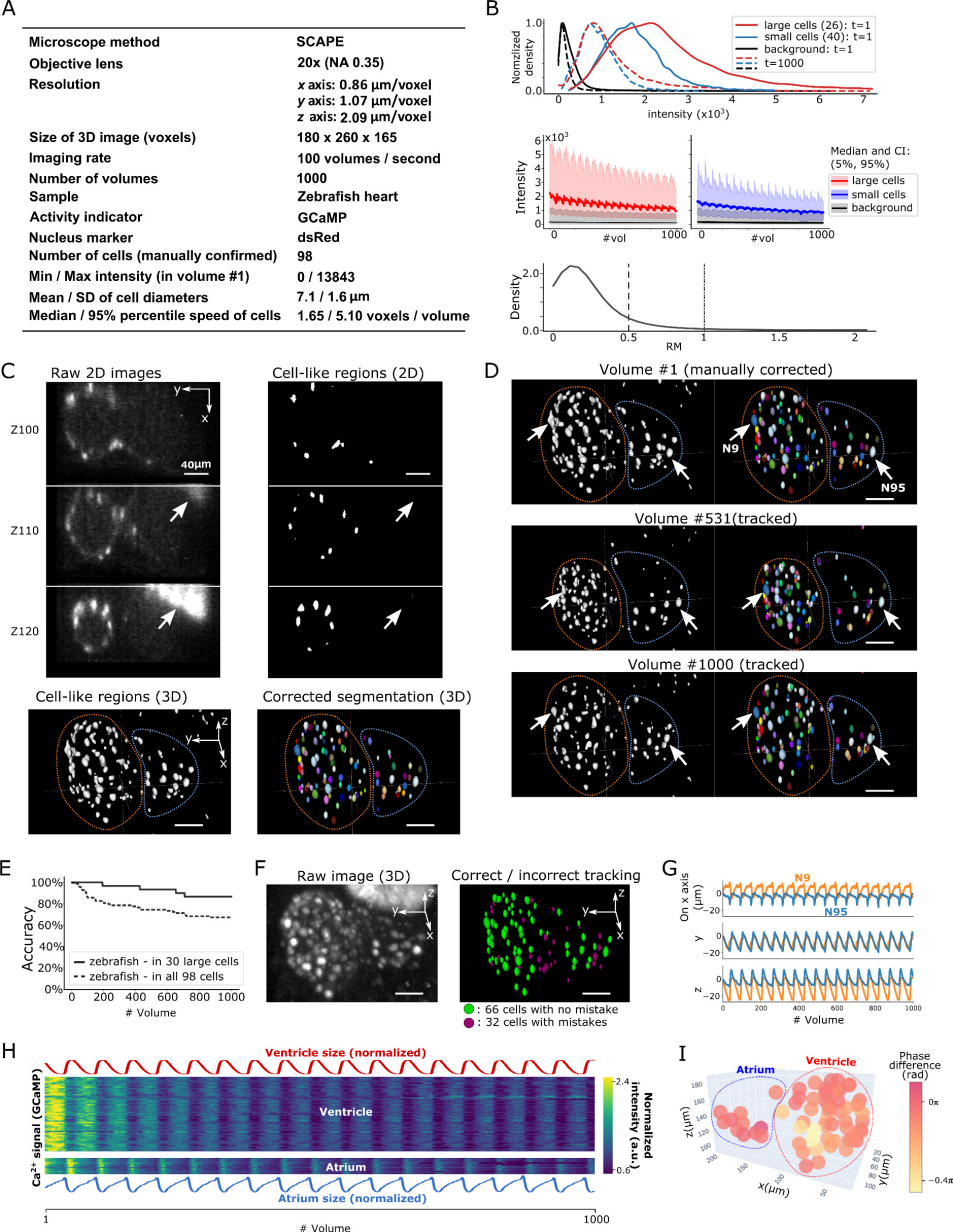
Results from tracking beating cardiac cells in zebrafish. (**A**) Experimental conditions. (**B**) Intensities and movements of the correctly tracked cells with large size (26 cells) or small size (40 cells). (**C**) 2D and 3D images and its segmentation result in volume #1. The large bright areas in Z110 and Z120 indicated by arrows are irrelevant for the tracking task, and our 3D U-net correctly classified them as background. Orange and blue outlines in bottom panels indicate the regions of the ventricle and atrium, respectively. (**D**) Tracking results. Tracked cells in volume #531 and #1000 are shown; we showed #531 because #500 looks similar to #1. Arrows indicate two correctly tracked representative cells in the ventricle and atrium. The sizes of the ventricle and atrium changed periodically (orange and blue outlines). (**E**) Tracking accuracy in 30 large cells or in all 98 cells through time. (**F**) The positions of 98 cells with/without tracking mistakes. (**G**) Movements of the two representative cells N9 and N95, indicating regular oscillations of cells in whole 3D space. (**H**) Dynamics of ventricle size, atrium size, and calcium signals in ventricle cells and atrium cells. The sizes of the ventricle and atrium cannot be directly measured, so we instead estimated them as size=sd(x)×sd(y)×sd(z), where *sd* is standard deviation (more robust than range of *x*, y, z), and *x*, *y*, *z* are coordinates of correctly tracked cells in the ventricle or in the atrium. To improve visibility, these sizes were normalized by size(normalized)=size/sd(size). Calcium signals (GCaMP) were also normalized by ${Ca}^{2+}(normalized)={Ca}^{2+}/mean({Ca}^{2+})$. (**I**) Phase differences between intracellular calcium dynamics and the reciprocal of segment sizes in ventricle and atrium were estimated. Here we used reciprocal of segment sizes because we observed the anti-synchronization relationships in (**H**). The phase differences were estimated using cross-correlation as a lag with the largest correlation. Most cells showed similar phase differences (mean = −0.110 π; standard deviation = 0.106 π). All scale bars, 40 µm.

To test the general applicability of the 3DeeCellTracker, we applied our method to the 3D + T images of a naturally beating heart in a zebrafish larva obtained at 100 volumes per second using a substantially different optical system, the SCAPE 2.0 system (Voleti et al., 2019; Figure 7A). The speed of image acquisition of this system is extraordinary relative to that of the spinning confocal system, which generally allows for the recording of ≤10 volumes/s. This dataset includes both large cells with stronger intensities that are easy to segment and small cells with weaker intensities that are difficult to segment (Figure 7B, top and middle). The photobleaching in the dataset made it challenging to detect and track the weak intensity cells in the later part of the imaging, because of the substantial overlap between the small cell signals and background signals (Figure 7B; Figure 7—figure supplement 1). This weak intensity is unavoidable because of the extremely quick scanning rate of the system (10,568 frames per second). In addition, the rapid beating of the heart caused relatively large movements of all the cells in the *x-y-z* directions (Figure 7B, bottom; Figure 7—video 1; see below), making cell tracking more challenging than that for worm #1–3, which predominantly moved in the *x-y* plane.

**Figure 7—figure supplement 1.**
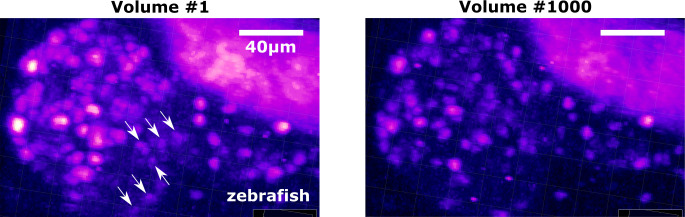
Examples of cells with weak intensities and photobleaching in the zebrafish dataset. All cells were visualized in 3D view. The left and right images are displayed under the same brightness/contrast setting; the right image (Volume #1000) was much darker than the left image (Volume #1) due to photobleaching. Arrows: example cells with weaker intensities that were more difficult to segment and track than the stronger ones. All scale bars, 40 µm.

**Figure 7—video 1.** dsRed signals in the zebrafish dataset. The intensity and movements of cell nuclei (dsRed) in the zebrafish dataset projected onto the 2D plane. The intensity of nuclei changed due to contraction and expansion of cells during beating.

**Figure 7—video 2.** Tracking results of the zebrafish dataset. The animation of beating cardiac cell regions (left) detected by 3D U-net and corresponding individual cells (right) in the zebrafish dataset tracked by our method.

After segmentation, we manually confirmed 98 cells in the first volume and tracked them automatically (Figure 7C and D; Figure 7—video 2). We found that among the 30 larger cells (size: 157–836 voxels) with higher intensities, 26 of them (87%) were correctly tracked in all 1000 volumes. Even though the smaller cells with lower intensities were more difficult to track, when including them, we still correctly tracked 66 out of 98 cells (67%) (Figure 7E and F). The tracked movements of two example cells showed regular oscillations in 3D space (*x*, *y*, and *z* axes; Figure 7G), consistent with the regular beating movement of the heart. It should be noted that we did not optimize the pipeline procedures for the zebrafish data, except for a few parameter changes (Table 2). Our results indicate that 3DeeCellTracker is also capable of analyzing images with rapid and dynamic movements in 3D space, obtained from a substantially different optical system.

We then used the tracking results to answer a biological question: What is the relationship between the intracellular calcium signals and the beating cycles of the heart? We extracted the calcium dynamics of the correctly tracked 66 heart cells, which co-express GECI as in the worm datasets, and analyzed the phases of activities in these cells relative to the beating cycles of the ventricular and atrial regions, respectively. As shown in Figure 7H and I, the calcium signals were largely synchronized with the beating cycles. Although a portion of the heart cells (32/98 total) was not correctly tracked and therefore not analyzed, this result is still remarkable because the tracked cells show the relationships between calcium dynamics and the natural heartbeats in vivo. Observation of this relationship was only made available by the developments of a state-of-the-art microscope system that can monitor 100 volumes per second and of our software pipeline that can correctly track large portions of the corresponding cell movements in 3D space.

### Tracking ~900 cells in a 3D tumor spheroid imaged with a two-photon microscope

We also tested our method on a dataset more related to biomedical application, namely a dataset of ~900 cells in a 3D multicellular tumor spheroid (MCTS) imaged with a two-photon microscope. 3D MCTS is increasingly being used for drug screening because of its similarity to tumor cells in vivo (Riedl et al., 2017). Therefore, the measurement of individual cell activities in 3D MCTS has become necessary, although tracking the movements of large numbers of cells in 3D + T images of 3D MCTS has presented a considerable challenge.

**Figure 8.**
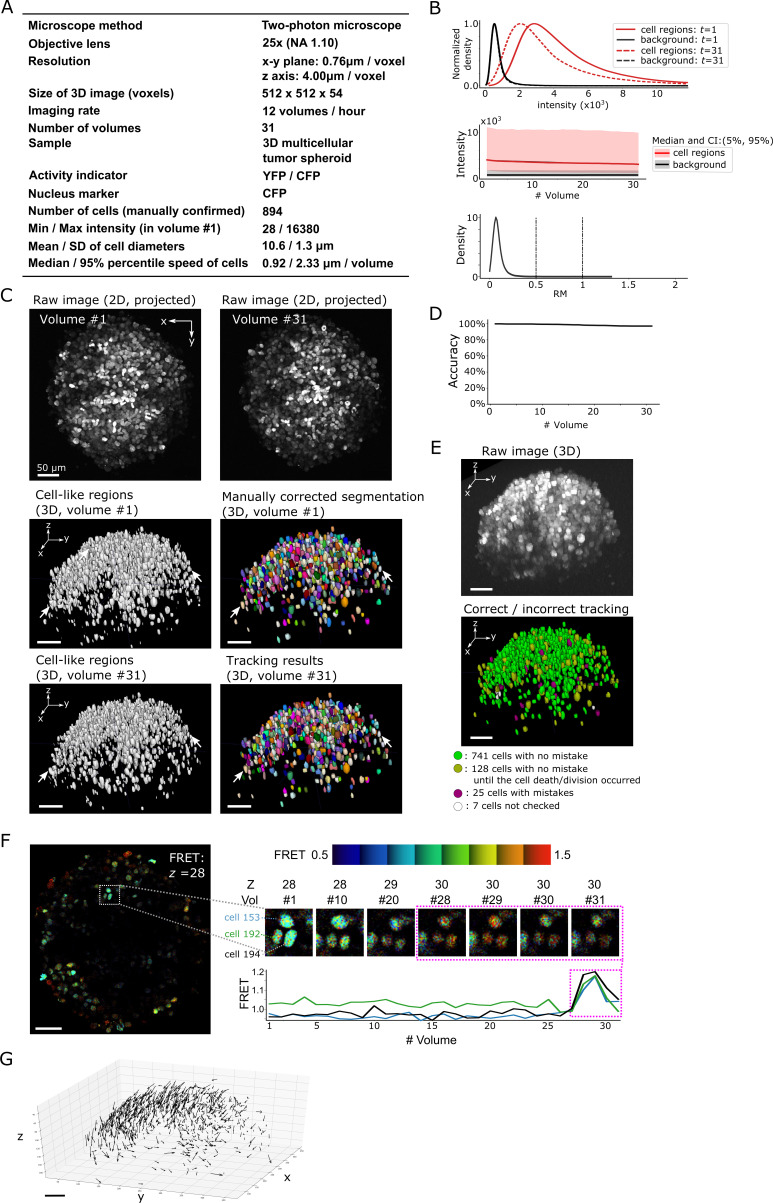
Results from tracking the cells in a 3D tumor spheroid. (**A**) Experimental conditions. (**B**) Intensities and movements. (**C**) Raw 2D images (top), cell-like regions (middle left and bottom left), segmented cells in volume #1 (middle right) and the tracking result in volume #31 (bottom right). (**D**) Tracking accuracy through time with a small decrease from 100% (volume #1) to 97% (volume 31). (**E**) The positions of cells with/without tracking mistakes. Seven cells (white) were not checked because segmentation mistakes were found in volume #1 in these cells during the confirmation of tracking. (**F**) An example of the extracted activity (FRET = YFP/CFP). Left: one layer of the FRET image at *z* = 28 in volume #1. The movements and intensities of three cells in the square are shown on the right side. The extracted activities of the three cells are shown in the graph. (**G**) Movements of the 741 cells that experienced neither tracking mistakes nor cell death/division. Arrows indicate the location changes from volume #1 to #31. All scale bars, 50 µm.

We obtained 3D + T images of a 3D MCTS cells expressing FRET type ERK sensor, EKAREV-NSL (Komatsu et al., 2011) using a two-photon microscope (see Materials and methods). This dataset shows normal distributions of intensities and movements but includes a much larger number of cells than either the worm brain or the zebrafish heart dataset (Figure 8A and B; Figure 8—video 1). Furthermore, cell division and death occurred during the imaging. Our method segmented and tracked 901 cells, of which we visually inspected the tracking results of 894 cells (the remaining seven cells were found to have segmentation errors in volume #1). We excluded the cells that experienced cell death or cell division after such events occurred, and found that 869 out of 894 cells (97%) were correctly tracked (Figure 8C–E, Figure 8—video 2). Using the tracking results, we extracted the ERK activity from the FRET signal. In three representative cells with cross-layer movements, we confirmed that the extracted signals correctly reflected intensity changes in the cells (Figure 8F). We also found that the spheroid as a whole moved downwards, although each cell moved in a different direction (Figure 8G).

**Figure 8—video 1.** CFP signals in the tumor spheroid dataset. The intensity and movements of cell nuclei (CFP) in the tumor spheroid dataset (3D view).

**Figure 8—video 2.** Tracking results of the tumor spheroid dataset. Upper: The cell regions detected by 3D U-net. Lower: Individual cells tracked by our method.

### Evaluation of the method under challenging conditions using degenerated datasets

In the assessments described in the preceding sections, we successfully analyzed multiple types of datasets that differ in terms of image resolution, signal-to-noise ratio, and types of cell movement etc. We then systematically evaluated the performance of our method (single mode) under a variety of conditions using a series of degenerated datasets obtained by modifying the worm #3 dataset. For a fair comparison, we used the same pre-trained 3D U-Net and the same manually corrected segmentation at *t* = 1 used on the original worm #3 dataset.

**Figure 9.**
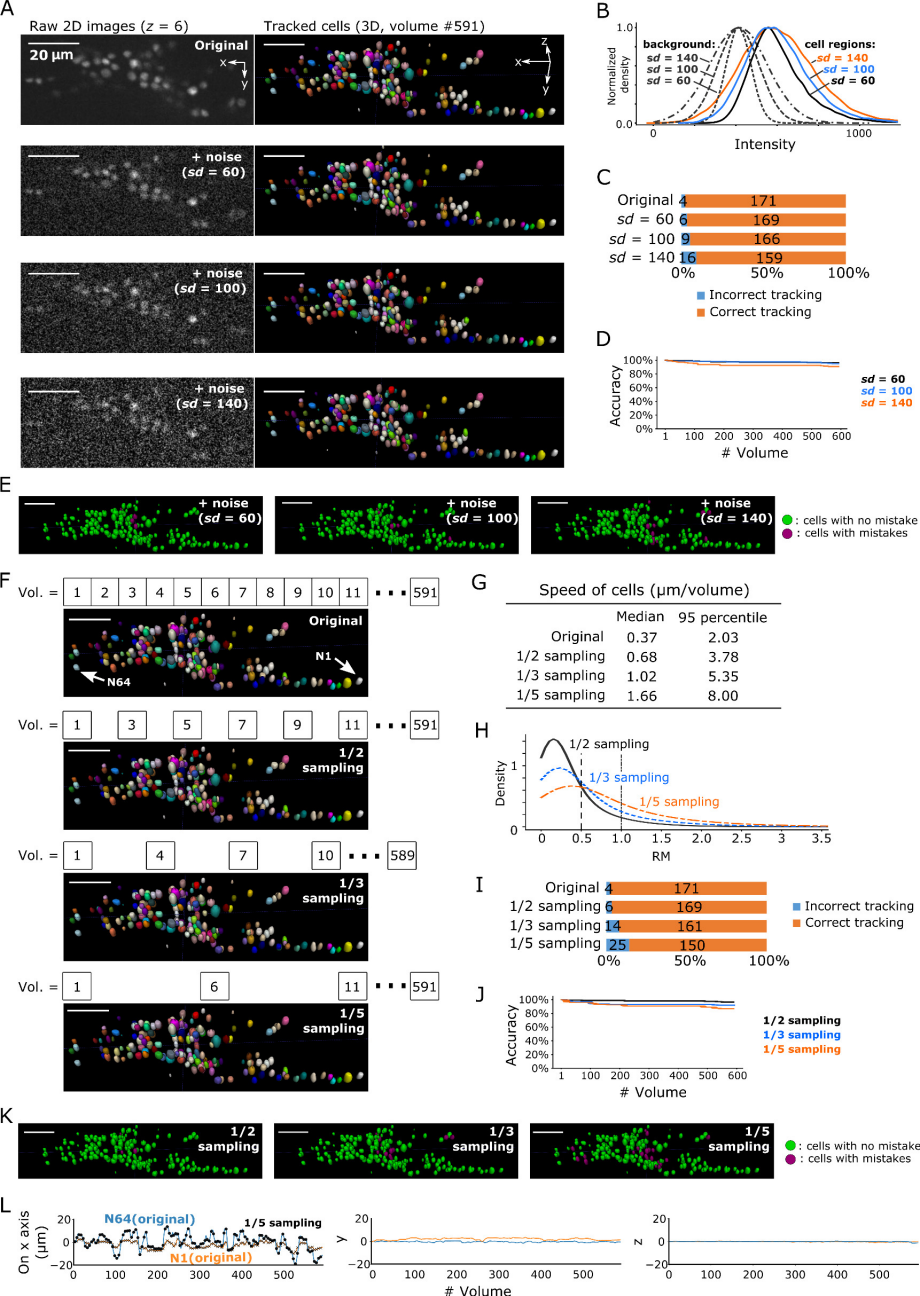
Robustness of our method in artificially generated challenging conditions. (**A**) Tracking results when Poisson noise was added to dataset worm #3. One layer (*z* = 6) of 2D images (left, volume #1) and the corresponding tracking results in the last volume were shown (right). (**B**) Distributions of intensities of cell regions (solid lines) and background (dashed lines) from images with added noise. All distribution curves were normalized so that their peaks have the same height. (**C**) Bar graph showing the numbers of incorrectly tracked and correctly tracked cells for the four noise levels. (**D**) Tracking accuracies of the datasets with added noise through time. (**E**) The positions of cells with/without tracking mistakes. The numbers of cells with correct/incorrect tracking are shown in panel C. (**F**) Tracking results in the last volume when sampling rates were reduced by removing intermediate volumes in dataset worm #3. Arrows indicate two representative cells. (**G**) Statistics of cell speed based on the tracking results of the 171 correctly tracked cells in the original images. The speeds in 1/5 sampling conditions are much larger than in the original conditions, which makes the cells more challenging to track. (**H**) Distributions of movements. (**I**) Bar graph showing the numbers of incorrectly tracked and correctly tracked cells for the four sampling rates. (**J**) Tracking accuracies of the downsampled datasets through time. (**K**) The positions of cells with/without tracking mistakes. The numbers of cells with correct/incorrect tracking are shown in panel I. (**L**) Movements of the two representative cells N64 and N1, indicating iterative expansion and contraction of the worm mainly in the *x*-axis. The movements between neighboring volumes after applying 1/5 sampling (black dots and crosses) are larger than the movements in the original dataset worm #3 (blue and orange lines). See Figure 3—figure supplement 3 and Figure 3—video 3 for tracking results in the original dataset worm #3. All scale bars, 20 µm.

A general difficulty in segmentation arises from images with low signal-to-noise ratios. Excessive noise can obscure the real cell signal, leading to incorrect segmentation and ultimately incorrect tracking. We tracked cells in three degenerated datasets with different levels of Poisson noise added to the original images: *sd* = 60, *sd* = 100, and *sd* = 140 (Figure 9A). The noise level in the original images in the non-cell regions was *sd* = 4.05 and the median intensity was 411, whereas the median intensity of cell regions was 567 with a 95% confidence interval of 430–934, indicating a tiny overlap between non-cell and cell regions (Figure 3—figure supplement 3B). In the *sd* = 60 condition, the intensities of non-cell and cell regions overlapped to a greater extent (Figure 9B), and image quality appeared poorer than that of the original image (Figure 9A). Nevertheless, our method achieved a low error rate (6/175 = 3%; Figure 9C–E). Even in the *sd* = 140 condition, in which the intensities overlapped extensively (Figure 9B) and the image quality was quite poor (Figure 9A), our method achieved an acceptable error rate (16/175 = 9%, that is, 91% correct; Figure 9C–E). Note that the tracking error rate was much lower than the segmentation error rate (16/175 vs 57/175, respectively; Figure 9—figure supplement 1), indicating that our FFN-based tracking method can compensate for errors in cell segmentation. Considering that the overlap of intensity distributions between cell regions and background in the degenerated datasets were much more severe than the overlap in the real datasets (Figure 9B and panel B in Figures 3, 6, 7 and 8), these results suggest that our method can robustly track cells in 3D + T images with severe noise.

**Figure 9—figure supplement 1.**
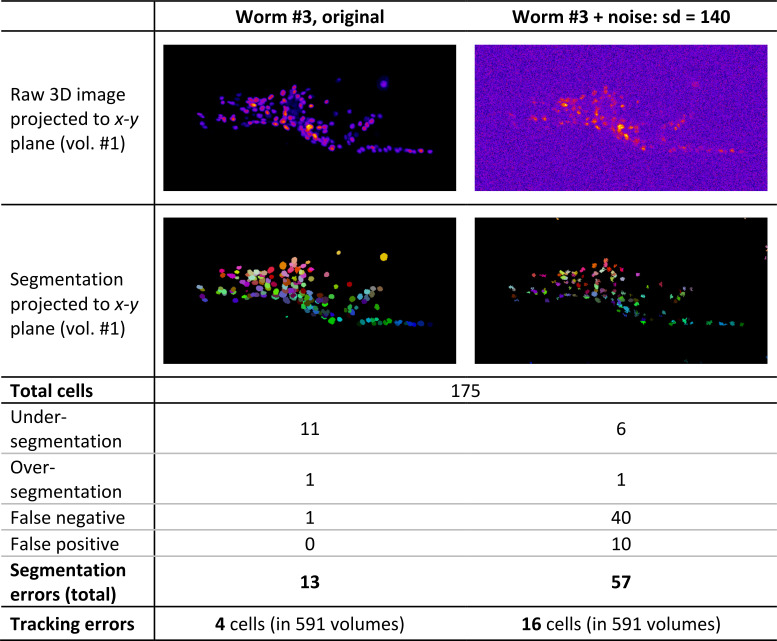
Errors in segmentation and tracking in two example image datasets. Numbers of the cells that were incorrectly segmented (in volume #1) and incorrectly tracked (in any volume) are shown. We manually checked the segmentation errors in two images. Compared to the number of segmentation errors (13 and 57), the number of tracking errors are much lower (4 and 16), implying that most incorrectly segmented cells were still correctly tracked. Note that segmentation errors were manually corrected in vol #1 but not in the following volumes.

**Figure 9—figure supplement 2.**
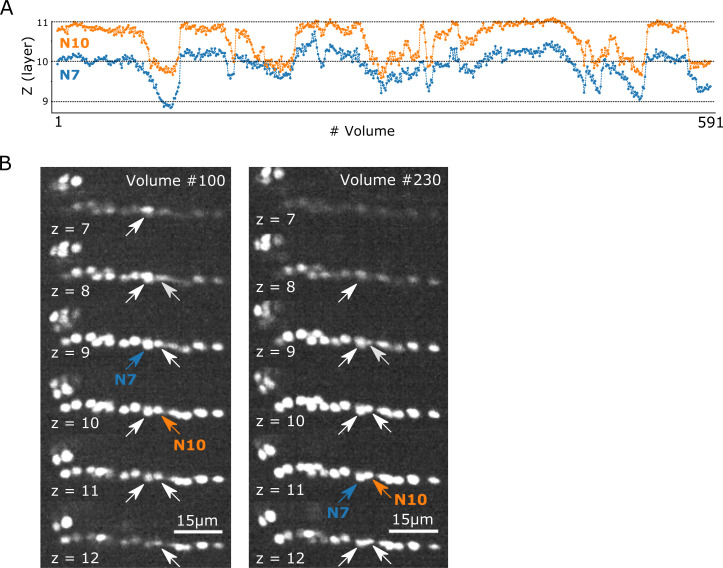
Cross-layer movements of two cells. (**A**) Examples of cross-layer movements of two cells in dataset worm #3. The positions of the centroids of each cell (z) were calculated as the average locations of the segmented regions corresponding to that cell. (**B**) The positions of the two cells (arrows) and their centroids (blue and orange) in raw images at volumes #100 and #230.

Another difficulty comes from large displacements of cells between volumes during tracking. We enhanced this effect by removing intermediate volumes in the worm's 3D + T images, and then tested the three datasets with 1/2, 1/3, and 1/5 of the volume of the original dataset (Figure 9F). As expected, when more volumes were removed, the movements of cells and the number of tracking errors increased (Figure 9G–L). Nevertheless, the error rate in the 1/3 vol condition was acceptable (14/175 = 8%, i.e. 92% correct), while the error rate in the 1/5 vol condition was relatively high (25/175 = 14%, i.e. 86% correct).

We then tested whether our method can track cell movements along the *z*-axis, in which the resolution is much lower than that in the *x-y* plane. In such conditions, 3D + T tracking is more challenging along the *z*-axis than in the *x-y* 2D plane. In the deforming worm and the tumor spheroid datasets, the resolution along the *z*-axis was approximately 1/10-1/5 of that in the *x-y* plane (panel A in Figure 3, Figure 3—figure supplements 1, 2 and 3, and Figure 8). We confirmed that 5, 16, 25, and 668 cells in worm #1a, #1b, and #3, and tumor spheroid datasets, respectively, showed cross-layer movements along the *z*-axis. Still, our method correctly tracked all of those cells. For example, while two cells in the worm #3 dataset exhibited multiple cross-layer movements (Figure 9—figure supplement 2), they were correctly tracked until the end the sequence. Furthermore, we also degenerated the zebrafish heart data to test whether our method can track dynamic 3D cell movements with unequal resolutions. Even when the zebrafish image resolution in the *z*-axis was reduced by sum binning with a shrink factor = 2, our method was able to correctly track a majority of the cells (80% of the 30 larger cells and 53% of all 98 cells; Table 3). Together, these results indicate that our method can correctly track cells in various conditions including severe noise, large movements and unequal resolution.

**Table 3.** Evaluation of large RM values in each dataset. We evaluated how challenging the tracking task is in each dataset using the metric ‘relative movement’ (*RM*). When *RM* ≥0.5, the cell cannot be simply tracked by identifying it as the closest cell in the next volume (see Figure 4A). When *RM* ≥1.0, the task becomes even more challenging. Note that large *RM* is just one factor making the segmentation/tracking task challenging. Lower image quality, photobleaching, three dimensional movements with unequal resolutions, lower coherency of cell movements, etc. also make tracking tasks more challenging. Some datasets share the same movement statistics because they are degenerated from the same dataset by adding noise or by modifying resolution. Also see Figures 4 and 10.

Dataset		Number of challenging movements				Accuracy
		With *RM* ≥ 0.5		With *RM* ≥ 1.0		
		Total	Per cell	Total	Per cell	
Worm #1a		110	**0.7**	0	**0.0**	**100%**
Worm #1b		0	**0.0**	0	**0.0**	**100%**
Worm #2		0	**0.0**	0	**0.0**	**100%**
Worm #3		6678	**39.1**	798	**4.7**	**98%**
Worm #3 + noise	sd = 60					**97%**
	sd = 100					**95%**
	sd = 140					**91%**
Worm #3 + reduced sampling	1/2	9965	**58.3**	2908	**17.0**	**97%**
	1/3	10255	**60.0**	4049	**23.7**	**92%**
	1/5	9117	**53.5**	4593	**26.9**	**86%**
Worm #4 (Freely moving)		20988	**318.0**	6743	**102.2**	**99.8% (ensemble mode)** **73% (single mode)**
Zebrafish	raw	5337	**80.9**	1185	**18.0**	**87%** (30 larger cells); **67%** (all 98 cells)
	binned					**80%** (30 larger cells); **53%** (all 98 cells)
Tumor spheroid		47	**0.1**	5	**0.0**	**97%**

### Challenging movement and its relationship with tracking error rate

**Figure 10.**
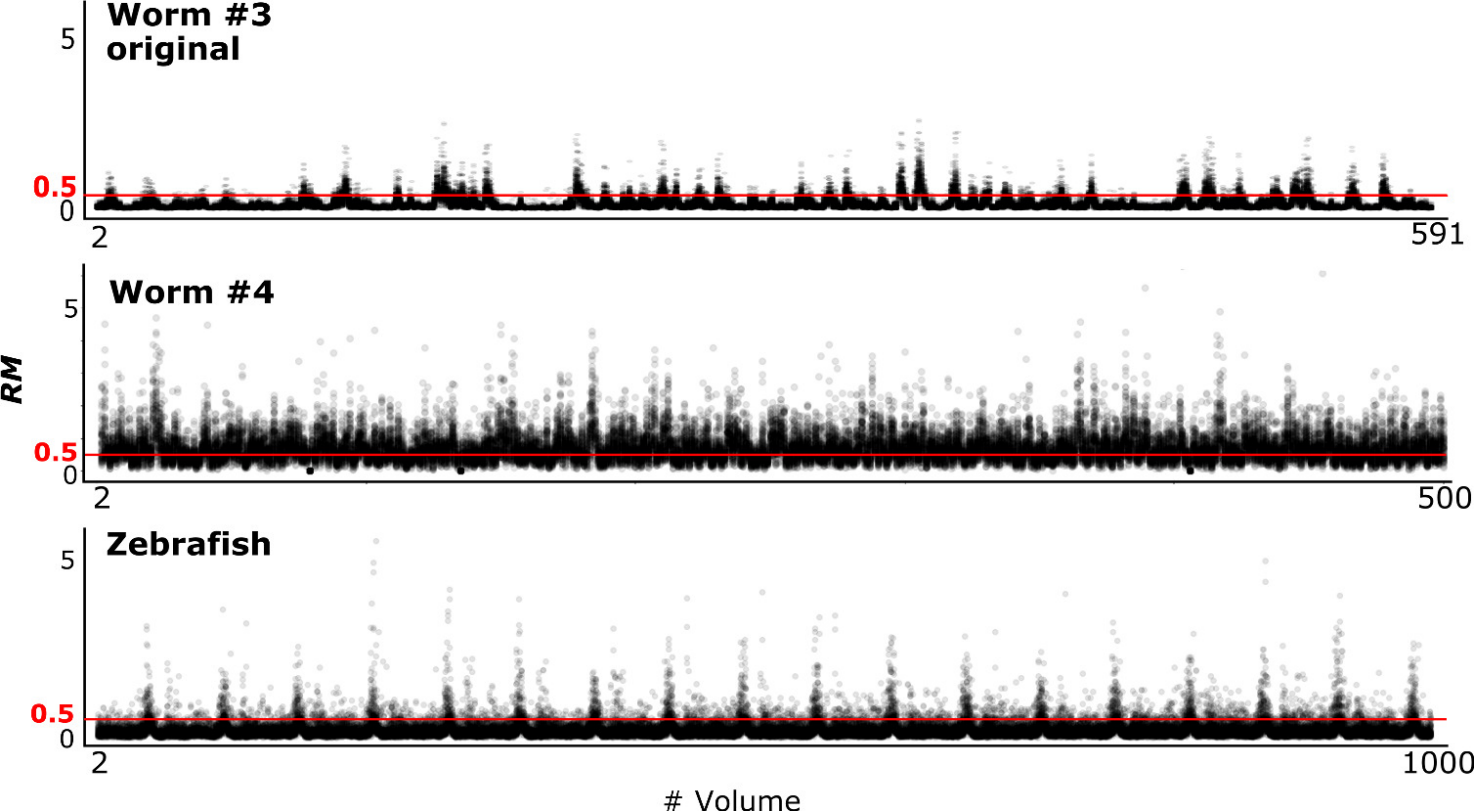
*RM* values at different time points in three datasets. Also see Figure 4 and Table 3.

To evaluate the tracking performance of our method on datasets with large movements, we summarized the *RM* values, which are listed in Table 3 and Figure 10 (see also Figure 4). Although many cell movements in the worm #3 and the zebrafish datasets had *RM* ≥0.5, most of the worm neurons and the larger cells in the zebrafish heart were correctly tracked by the single mode. In addition, our program with the ensemble mode achieved 99.8% tracking of the neurons of a ‘straightened’ freely moving worm while many cell movements in this dataset had RM ≥1. These results indicate that our method is capable of analyzing images with challenging displacements.

**Figure 10—figure supplement 1.**
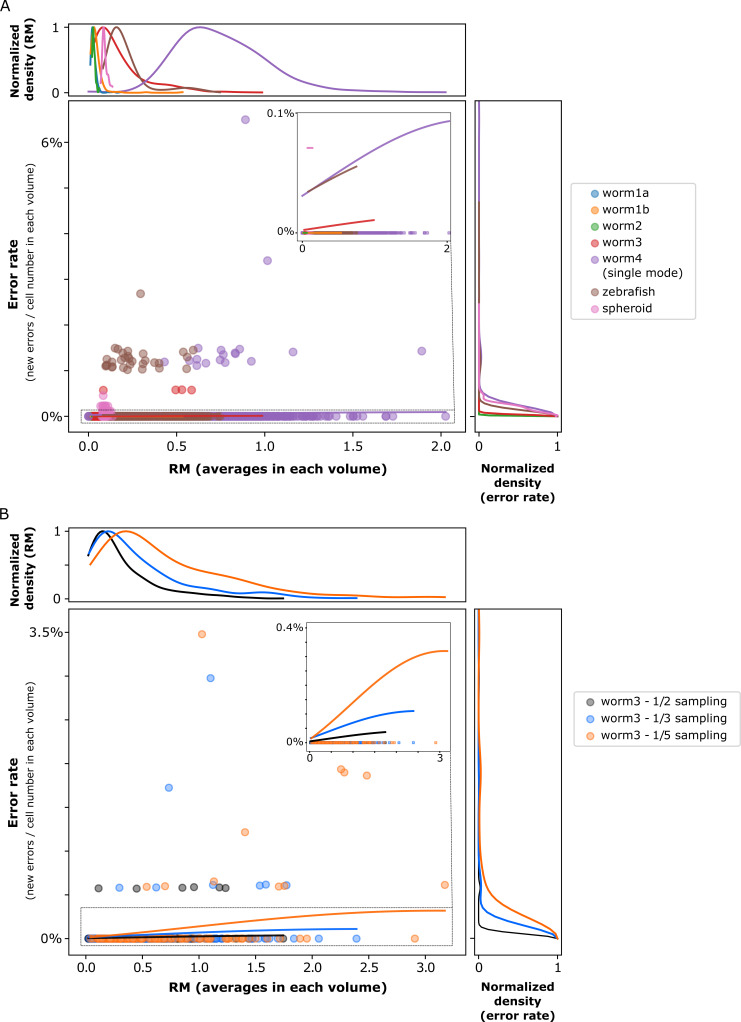
The relationship between movements and the error rate of our method. (**A**) The relationship in the real datasets. (**B**) The relationship in the downsampled worm #3 datasets. The *RM* is the averaged *RM* in each volume. The error rate is the number of new errors divided by the number of correctly tracked cells until the last volume. We fitted regression lines using the kernel ridge regression with RBF kernel. The top and right parts show the marginal distributions of *RM* and error rate, respectively.

**Figure 10—figure supplement 2.**
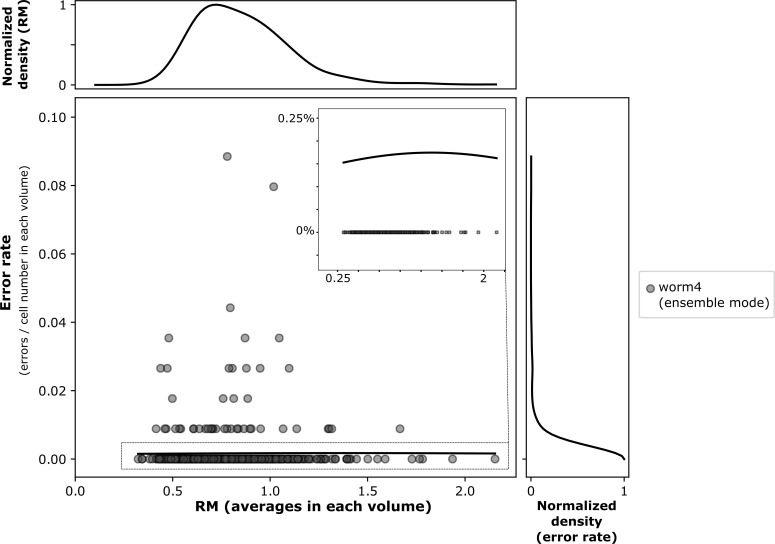
The relationship between movements and the error rate of our method in ensemble mode. The result is based on the freely moving worm dataset (worm #4).

To investigate the relationships between the cell movements and tracking error rate, we drew a scatter plot of the averaged *RM* and corresponding error rate for each volume (Figure 10—figure supplement 1). The results indicated by the regression lines suggest positive correlations between the *RM* and error rate, although the correlation trends appear to differ by dataset, implying that movement is not the only factor affecting the error rate. We also drew a scatter plot and regression line for the worm #4 dataset tracked with the ensemble mode (Figure 10—figure supplement 2). The result suggests that the error rate is not correlated with movement, perhaps because in ensemble mode the cell positions are predicted from multiple different volumes, and therefore movement from a previous volume is not closely connected with the error rate.

### Comparison of the tracking accuracies of our and previous methods

To further assess the capabilities of our method, we compared the tracking performance of our method with that of two other state-of-the-art methods for cell tracking. The first was DeepCell 2.0, a newer version of DeepCell, which is a pioneer in segmenting and tracking cells in 2D + T images using deep learning (Bannon et al., 2018). Unlike our method, DeepCell 2.0 has been primarily tested on images that include cell divisions, birth and death, but not on images of deforming organs. The second was the software developed by Toyoshima et al., which does not use a deep learning technique, but has achieved a higher accuracy in both segmenting and tracking of worm whole brain datasets than any other previous methods (Toyoshima et al., 2016).

**Figure 11.**
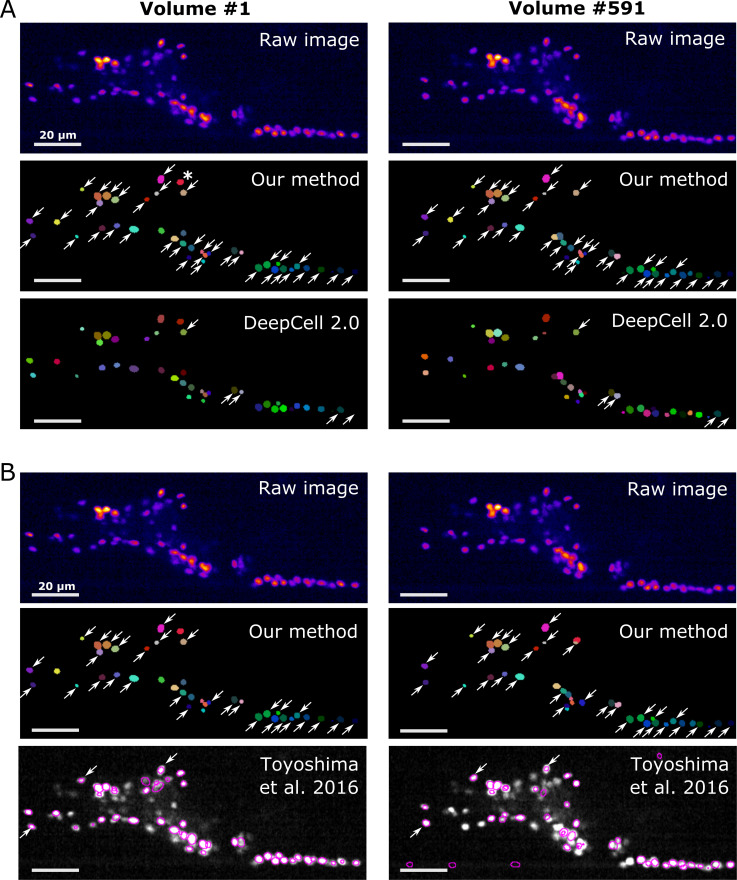
Comparison of the tracking results between our method, DeepCell 2.0, and Toyoshima et al., 2016. (**A**) Comparison with DeepCell 2.0 tested using the layer *z* = 9 in worm #3. The raw images (top) are displayed using the pseudocolor ‘fire’. Colors in the tracking results (middle and bottom) indicate the initial positions (volume #1, left) and tracked positions of cells (volume #519, right). Note that the spatial patterns of cell colors in the initial and last volumes are similar in our method (middle) but not in DeepCell 2.0 (bottom). Arrows indicate all the correctly tracked cells in our method and in DeepCell 2.0. Cells without arrows were mistracked. The asterisk indicates a cell whose centroid moved to the neighboring layer (*z* = 10) and thus was not included in the evaluation. Also see Figure 11—video 1 and Table 4. (**B**) Comparison with Toyoshima’s software tested using all layers in worm #3 with 1/5 sampling rate. For demonstration, we only show tracking results at *z* = 9. Again arrows indicate all the correctly tracked cells in our method and in Toyoshima’s software. Because Toyoshima’s software is not able to label the tracked cells using different colors, all cells here are shown by purple circles. Some cells were not marked by circles because they were too far from the centroids of the cells (in other layers). See also Table 4. All scale bars, 20 µm.

On our worm brain datasets, DeepCell 2.0 tracked ~10% of cells properly (Figure 11A, Figure 11—figure supplement 1A, Figure 11—video 1, and Table 4); this is probably because the tracking algorithm of DeepCell 2.0 is optimized for the movements associated with cell divisions, but not for quickly deforming organs. Toyoshima’s method tracked ~90% of the original worm #3 neurons but only ~10% of the degenerated ‘worm #3, 1/5 sampling’ (Figure 11B, Figure 11—figure supplement 1B, and Table 4). When tested on the zebrafish dataset, Toyoshima's method was able to detect only 76 cells, and missed some weak-intensity cells, performing worse than our method (detected 98 cells). Of the detected 76 cells, 21 were incorrectly tracked from the first volume, probably because their method automatically re-fits the cell shape using a Gaussian distribution after segmentation, which can lead to a failure when fitting weak-intensity cells. Their tracking accuracy was also lower than ours (Table 4). In addition, our method was found comparable to or more efficient in terms of runtime than DeepCell 2.0 and Toyoshima's methods (Table 5). These results suggest that our method is more capable of tracking cells in 3D + T images than previous methods.

**Figure 11—figure supplement 1.**
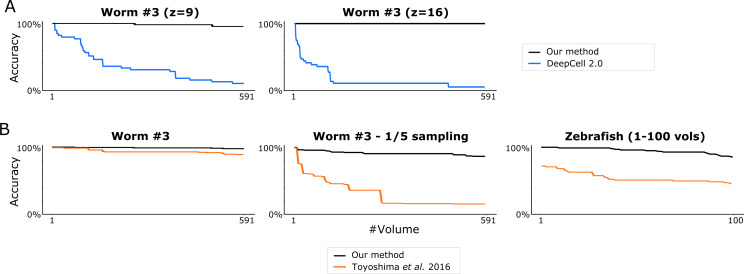
Comparision of the tracking accuracies between our method and two previous methods. (**A**) Tracking accuracies by DeepCell 2.0 and by our method for two 2D image datasets. (**B**) Tracking accuracies by Toyoshima et al., 2016 and by our method for three 3D image datasets. For the zebrafish dataset, Toyoshima ‘s method showed low accuracy from the first volume because of segmentation errors.

**Figure 11—video 1.** Tracking results of a 2D + T image (*z* = 9 of worm #3) using our method and DeepCell 2.0. The tracked cells are shown in different colors. In our method, most cells were correctly tracked and their colors did not change (top). In DeepCell 2.0, most cells were incorrectly tracked and their colors changed during the tracking (bottom).

**Table 4.** Tracking accuracy of our method compared with DeepCell 2.0 and Toyoshima et al., 2016. We evaluated the tracking accuracy in two 2D + T image datasets for the comparision with DeepCell 2.0, because DeepCell 2.0 currently cannot process 3D + T images. We also evaluated the tracking accuracy in three 3D + T image datasets for comparision with Toyoshima et al., 2016. For the zebrafish dataset, we only processed the initial 100 volumes because Toyoshima’s software requires a very long processing time (Table 5).

Dataset	Method	Correct tracking	Accuracy
Worm #3 (z = 9)	**Our method**	**37 (in 39 cells)**	**95%**
	DeepCell 2.0	5 (in 39 cells)	13%
Worm #3 (z = 16)	**Our method**	**36 (in 36 cells)**	**100%**
	DeepCell 2.0	2 (in 36 cells)	6%
Worm #3	**Our method**	**171 (in 175 cells)**	**98%**
	Toyoshima et al., 2016	157 (in 175 cells)	90%
Worm #3: 1/5 sampling	**Our method**	**150 (in 175 cells)**	**86%**
	Toyoshima et al., 2016	21 (in 175 cells)	12%
Zebrafish (100 volumes)	**Our method**	**84 (in 98 cells)**	**86%**
	Toyoshima et al., 2016	35 (in 76 cells)	46%

**Table 5.** Runtimes of our method and two other methods. We tested the runtime of our method and two other methods on our desktop PC.

Dataset	Method	Runtime per volume	Comments
Worm #3	**Our method**	**~38 s**	21 layers
	DeepCell 2.0 (for 2D)	**~**33 s	= 1.57 s x 21 layers
Worm #3	**Our method**	**~38 s**	
	Toyoshima et al., 2016	**~**140 s	
Zebrafish	**Our method**	**~1 min**	
	Toyoshima et al., 2016	**~**15 min	

## Discussion

Tracking biological objects in 3D + T images has proven to be difficult, and individual laboratories still frequently need to develop their own software to extract important features from the images obtained using different optical systems and/or imaging conditions. Moreover, even when identical optical systems are used, the optimization of many parameters is often required for different datasets. To solve these problems, we have developed a deep learning-based pipeline, 3DeeCellTracker, and demonstrated that it can be flexibly applied to divergent datasets obtained under varying conditions and/or different qualities. We analyzed multiple image series of worms, zebrafish, and tumor spheroids, which differed in terms of nuclear marker, intensity level, noise level, numbers of cells per image, image resolutions and sizes, imaging rates, and cell speed. Notably, we showed that our method successfully tracked cells in these datasets under challenging conditions, such as large movements (see Figure 10, Table 3 and Materials and methods), cross-layer movements (Figures 6G and 7G, and Figure 9—figure supplement 2), and weak intensity conditions (Figure 7B, Figure 7—figure supplement 1, Figure 7—video 1). In addition, our method outperformed two previous state-of-the-art cell tracking methods, at least in deforming organs under challenging imaging conditions (Figure 11, Figure 11—figure supplement 1, and Table 4), while the other methods are likely more suited for other conditions. Furthermore, our method is comparable to or more efficient in runtime than previous methods (Table 5 and Materials and methods). Running in ensemble mode on a desktop PC, our method tracked the neurons of a ‘straightened’ freely moving worm with high accuracy, which required a computing cluster with up to 200 cores in the previous study (Nguyen et al., 2017).

We consider that the high accuracy and robustness of our method is based on its use of FFN, a deep learning technique, together with its post-processing methods for tracking (Figure 2—figure supplement 3). As mentioned above, although deep learning techniques have been predicted to exhibit superior performance in 3D cell tracking, they had not been used to date because of the difficulty in manually preparing large quantities of training data, especially for 3D + T images. To solve this problem, we generated a synthetic training dataset via artificial modification of a single volume of worm 3D cell positional data, which produced excellent performance by our method. These results demonstrate that the deep network technique can be used for cell tracking by using an synthetic point set dataset, although the procedures for generating the dataset are simple.

Not only is our method flexible and efficient, it can be easily used by researchers. For example, our method worked well in all the diverse conditions tested with only minor modifications. Notably, under constant imaging conditions, our method can be directly reused without modifying any parameters except for the noise level (Tables 1 and 2), making it convenient for end-users. This differs from conventional image processing methods, in which slight differences in the obtained data, such as in light intensity, resolution, the size of the target object etc, generally require the re-setting of multiple parameters through trial-and-error. Even when imaging conditions are substantially changed, our method requires only a few modifications, primarily in the segmentation process: (1) modifying the structure of the 3D U-Net (this step can be skipped because a 3D U-Net of the same structure can be adapted to a new dataset by re-training; see Materials and methods); (2) re-training the 3D U-Net; and (3) modifying parameters according to the imaging conditions (see Tables 1 and 2 and the ‘Guide for parameters.md’ file in https://github.com/WenChentao/3DeeCellTracker). For re-training the 3D U-Net, manual annotation usually takes 2–3 hr for 150–200 cells, and the network can be automatically trained in 1–2 hr on our desktop PC with a single GPU. The FFN for tracking generally does not require re-training. The number of parameters to be manually determined is much smaller in our method than in conventional methods (Table 1) due to its use of deep learning. The parameters can be quickly modified (within 1 hr) following the guide we have provided in the GitHub repository.

Nevertheless, our method can be improved in two ways: more reliable tracking and a simplified procedure. Tracking reliability can be affected by large movements, weak intensities, and/or photobleaching. As revealed in our results (Figure 6), large movements such as in a freely moving worm can be resolved using the ensemble mode, which borrows the idea from ensemble learning in machine learning, that is using the average of multiple predictions to reduce the prediction error (Polikar, 2009). A similar idea, matching cells using multiple reference volumes and a clustering method instead of averaging, was applied in the previous study (Nguyen et al., 2017), suggesting that ensemble learning is a good approach to resolving challenging movements. On the other hand, the problem of weak intensity and photobleaching remains to be solved. One possible approach would be to normalize the intensities to obtain similar images over time, although doing so might not be easy.

To further simplify the entire procedure, we contemplate developing new network structures that combine additional steps. The U-Net and 3D U-Net are networks for semantic segmentation, which classify each voxel as a specific category, that is as either a cell or a non-cell region. Beyond this, networks have been designed to achieve instance segmentation by further separating objects in the same category into individual objects, eliminating the need to use a watershed for separating connected cells. Although recent advances have been made in these architectures, the focus is still on segmenting common objects in 2D images (Liang et al., 2016; Romera-Paredes and Torr, 2016; He et al., 2017). We suggest that instance segmentation is a possible approach for simplifying and improving cell segmentation in future studies. Another possible area for improvement is the use of FFN for tracking. By further improving the FFN structure and using more training data, the network should be able to generate more accurate matches that can directly be used for tracking cells without point set registration.

We developed 3DeeCellTracker mainly using semi-immobilized worm datasets. However, it also successfully processed 3D + T images of a zebrafish dataset obtained using the SCAPE 2.0 system (Voleti et al., 2019). This system is quite different from the spinning disk confocal system used for worm datasets in resolution, *z*-depth, and applied optical sectioning principle (Bouchard et al., 2015). While SCAPE is an original and outstanding method for enabling ultra high-speed 3D + T image acquisition, it had been difficult to obtain or develop software that can efficiently process the 3D + T images produced by the system. In this study we tracked 3D + T images obtained from the SCAPE system by simply modifying a few parameters, which allowed us to obtained an acceptable result (87% of large cells correctly tracked). Considering that the lower performance relative to other datasets might have arisen from the difficulty in segmenting the smaller, low-intensity cells (Figure 7B, the upper and the middle panels), the result may be improved by further optimization of the segmentation.

We also successfully tracked a large number of cells (~900) in a 3D MTCS monitored using a two-photon microscope, a result that further supports the wide applicability of our method. Our method cannot track cells that are dividing or fusing, or many cells that enter the field of view during the recording. This is because it operates under the assumption that each cell has a unique corresponding cell in another volume in order to match cells with large movements. To handle cells with division, fusion, or entry, it will be necessary to integrate our algorithms with additional algorithms.

In summary, we have demonstrated that 3DeeCellTracker can perform cell segmentation and tracking on 3D + T images acquired under different conditions. Compared with the tracking of slowly deforming cells in 2D + T images, it is a more challenging task to track cell nuclei in a semi-constrained/freely moving worm brain, beating zebrafish heart, or 3D tumor spheroid, all of which undergo considerable movements in 3D space. We consider this to be the first report on a pipeline that efficiently and flexibly tracks moving cells in 3D + T images from multiple, substantially different datasets. Our method should enable the segmentation and tracking of cells in 3D + T images acquired by various optical systems, a task that has not yet been performed.

## Materials and methods

**Key resources table:** 

Reagent type (species) or resource	Designation	Source or reference	Identifiers	Additional information
Strain, strain background (*Caenorhabditis elegans*, hermaphrodite)	KDK54165 AML14	This paper Nguyen et al., 2016	RRID:WB-STRAIN:KDK54165 RRID:WB-STRAIN:AML14	*lite-1(xu7);oskEx54165[rab-3p::GCaMP5G-NLS, rab-3p::tdTomato] ;wtfEx4[rab-3p::NLS::GCaMP6s, rab-3p::NLS::tagRFP]*
Cell line (*Homo sapience*)	HeLa cells	Riken Cell Bank	RCB0007, RRID:CVCL_0030	
Transfected construct	EKAREV-NLS	Komatsu et al., 2011		FRET-type ERK sensor
Software, algorithm	3DeeCellTracker ImageJ ITK-SNAP IMARIS	This paper ImageJ ITK-SNAP IMARIS	RRID:SCR_003070 RRID:SCR_002010 RRID:SCR_007370	

### Computational environment

Our image processing task was performed on a personal computer with an Intel Core i7-6800K CPU @ 3.40 GHz x 12 processor, 16 GB of RAM, and an Ubuntu 16.04 LTS 64-bit operating system. We trained and implemented the neural networks with an NVIDIA GeForce GTX 1080 GPU (8 GB). The neural networks were constructed and implemented using the Keras high-level neural network API (https://keras.io) running on top of the TensorFlow machine-learning framework (Google, USA). All programs were implemented within a Python environment except for the image alignment, which was implemented in ImageJ (NIH; RRID:SCR_003070), and the manual labeling, manual correction and manual confirmation, which were implemented in ITK-SNAP (RRID:SCR_002010; http://www.itksnap.org) or IMARIS (Bitplane, UK; RRID:SCR_007370). Instead of ITK-SNAP and IMARIS, one can use napari (https://napari.org) in the Python environment.

### Pre-processing

Step 1: Because 2D images were taken successively along the *z* axis, rather than simultaneously, small or large displacements could exist between different layers of a 3D volume. Ideally, this should be compensated for before the segmentation procedure. Using the StackReg plugin (Thevenaz et al., 1998) in ImageJ (NIH), we compensated for the displacements by using rigid-body transformations to align each layer with the center layer in worm #1 and #2 datasets. However, we did not apply this alignment in worm #3, #4, zebrafish, and tumor spheroid datasets but still obtained acceptable results, indicating this step may be skipped.

Step 2: Cells in the same image could have very different intensities, and detecting weak cells is generally difficult. To solve this problem, we applied local contrast normalization (Goodfellow et al., 2017) through a sliding window (27 × 27 × 3 voxels) so that all cells had similar intensities. This normalization was applied to the nucleus marker images only for tracking and did not affect the calculation of the signal intensities for either red fluorescent protein or GECI.

### 3D U-Net

We used 3D U-Net structures similar to that shown in the original study (Çiçek et al., 2016). The network received a 3D image as input and generated a 3D image of the same size with values between 0 and 1 for each voxel, indicating the probability that the voxel belonged to a cell region (Figure 2—figure supplement 1). We used different structures of 3D U-Net for different imaging conditions, in order to capture as much information as possible of each cell within the limitations of the GPU memory. Such modification of the U-Net structures is preferred but not necessary. The same structure can be reused on different datasets because of the flexibility of deep learning methods. The 3D U-Net structure shown in Figure 2—figure supplement 1A was used on our datasets worm #1 and worm #2, which have identical resolution, but we also successfully used the same structure in the binned zebrafish dataset (see below), which had very different resolution. For dataset worm #3, which had a lower resolution, we reduced the number of maxpooling and upsampling operations so that each voxel in the lowest layers corresponds to similar sizes as in the datasets worm #1 and worm #2. We also reduced the sizes of the input, output, and intermediate layers because of the lower resolution. As the smaller structure occupied less GPU memory, this allowed us to increase the number of convolutional filters on each layer so that the capacity of the network was increased (see Figure 2—figure supplement 1B). For the zebrafish dataset, although it has even lower resolution in the *x* and *y* axes, because the sizes of zebrafish cardiac cells are larger than worm neurons, we used the same number of maxpooling and upsampling operations as in datasets worm #1 and worm #2. We adjusted the sizes of layers in the *x*, *y*, and *z* dimensions to a unified value (=64) because the resolution in the three dimensions are not very different in the zebrafish dataset (Figure 2—figure supplement 1C). For simplicity, we reused the structures A and B in Figure 2—figure supplement 1 for worm #4, tumor spheroid and binned zebrafish dataset (see Table 2).

The U-Net can be trained using very few annotated images (Ronneberger et al., 2015). In this study, we trained six 3D U-Nets: (1) for datasets worm #1 and #2, (2) for dataset worm #3, (3) for freely moving dataset worm #4, (4) for the zebrafish dataset, (5) for the dataset tumor spheroid, and (6) for the binned zebrafish dataset. Each 3D U-Net used one 3D image for training. Note that, although datasets worm #1 and #2 are substantially different with respect to signal intensity and cell movements, the same trained 3D U-Net was used. The image was manually annotated into cell regions and non-cell regions using the ITK-SNAP software (http://www.itksnap.org). We used the binary cross-entropy as the loss function to train the 3D U-Net. Because the raw image sizes were too large (512 × 1024 × 28, 256 × 512 × 20, 180 × 260 × 165, etc.) for computing in the GPU, we divided the raw images into small sub-images that fit the input sizes of the three 3D U-Nets structures (160 × 160 × 16, 96 × 96 × 8, or 64 × 64 × 64), and combined the cell/non-cell classifications of sub-images to form a final classification of the whole image. To improve the 3D U-Net performance, we increased the training data by data augmentation: We applied random affine transformations to the annotated 3D images by ‘ImageDataGenerator’ class in Keras. The affine transformation was restricted in the *x-y* plane but not in the *z*-direction because the resolution in the *z*-direction is much lower than that in the *x-y* plane for worm datasets and the tumor spheroid dataset (see panel A in Figure 3, Figure 3—figure supplements 1, 2 and 3, and Figure 8). Although the zebrafish dataset has similar resolutions in the *x*, *y*, and *z* directions, we applied the same affine transformation for simplicity. We trained the U-net for datasets worm #1 and #2 using a 3D image from another dataset independent of #1 and #2 but obtained under the same optical conditions, and its classifications on datasets worm #1 and #2 were still good (panel C in Figure 3, Figure 3—figure supplements 1 and 2), indicating a superior generalization ability of the 3D U-net. Because only one dataset is available for the specific resolutions of dataset worm #3, #4, the zebrafish heart, and the tumor spheroid, we trained the 3D U-Net by using the first volume of 3D + T images of each dataset and then applied the 3D U-Net to all the following 3D images of the datasets.

### Watershed

The 3D U-Net generated probability outputs between 0 and 1, which indicated the probability that a voxel belonged to a cell-like region. By setting the threshold to 0.5, we divided the 3D image into cell-like regions (>0.5) and non-cell regions (≤0.5). The cell-like regions in the binary images were further transformed into distance maps, where each value indicated the distance from the current voxel to the nearest non-cell region voxel. We applied a Gaussian blur to the distance map to smooth it, and searched for local peaks which were assumed to be cell centers. We then applied watershed segmentation (Beucher and Meyer, 1993), using these centers as seeds. Watershed segmentation was applied twice; the first application was 2D watershed segmentation for each *x-y* plane, and the second application was 3D watershed segmentation for the entire 3D space. Two segmentations were required because the resolutions in the *x-y* plane and the *z*-dimension differed.

### Feedforward network: architecture

An initial matching, that is, a set of correspondences between cells in two temporally adjacent volumes, is the first step for cell tracking in our pipeline and critical for the final tracking accuracy. The correspondences can be estimated based on the relative cell positions, assuming that these positions do not substantially change even during organ deformation. By comparing the similarity between the relative positions of two cells in different volumes, we can determine whether they are the same cells.

One conventional method to represent relative positions is fast point feature histograms (FPFH) (Rusu et al., 2009). The PR-GLS study (Ma et al., 2016) successfully used the FPFH method to match artificial point set datasets. However, we found that FPFH yielded a poor initial match for the datasets considered in this study (Figure 2—figure supplement 3A), perhaps because of the sparse distribution of the cells. We thus designed a three-layer feedforward network (FFN) to improve the initial match (Figure 2B). The three-layer structure generated good match results comparable with those of more complex structures with four or six layers. By comparing the representations between two points, the network generated a similarity score between two cells. The initial matching based on the similarity score by the FFN was more accurate than that achieved by the FPFH method (Figure 2—figure supplement 3B). Our FFN + PR-GLS approach generated more accurate predictions of cell positions than FPFH + PR-GLS and the simple method of affine alignment (using an implementation of the Coherent Point Drift Algorithm: https://github.com/siavashk/pycpd) (Figure 2—figure supplement 3C and D).

In our FFN, the input of the network contained the position information of two points. Each point was represented by the normalized positions of the 20 nearest neighboring points (Figure 2B). The 20 nearest neighbor positions ($\vec{d_{1}},\;\vec{d_{2}},\;\ldots ,\;\vec{d_{20}}$) were given by 3D vectors because they were extracted from the 3D image. To normalize the points, each of the 20 positions was divided by the mean distance d ($d=\frac{1}{20}\sum_{k=1}^{20}\left|\vec{d_{k}}\right|$). The normalized positions were then sorted by their absolute values in ascending order. Finally, the mean distance d was included as the last value, so each point was represented by a 61D vector.

We utilized the first fully connected layer to calculate the learned representation of the relative positions of each point as a 512D vector (Figure 2B, the first hidden layer after the input). We then applied a second fully connected layer on these two 512D vectors to compare the representations of the two points. The resulting 512D vectors (the second hidden layer after the input) were processed by a third fully connected layer to obtain a single similarity score between 0 and 1, which indicated the probability of two points originating from the same cell. We matched the two points with the highest scores in two different volumes, ignored these two points, and matched the next set of two points with the highest scores. By repeating this process, we obtained an initial match (Figure 2C, panel 4–1).

### Feedforward network: training

In this study, we only trained one FFN based on one original image of dataset worm #3 and used the network in all the datasets including worms, zebrafish and tumor spheroid. To train the network, we first performed segmentation on a single volume of dataset worm #3 and obtained a point set for the centers of all cells. Because we required a large number of matched point sets for training, and because manually matching point sets is time-consuming and impractical, we created an synthetic training dataset by applying random affine transformations to the point set described above and adding small random movements to each point according to following equations:

(1) $$\vec{x^{\prime }}=A\vec{x}+\vec{\varepsilon_{1}}+\vec{\varepsilon_{2}}$$

Also see Figure 2—figure supplement 2A for an illustration. Here $\vec{x}$ is the mean-removed 3D position {$x_{1}$, $x_{2}$, $x_{3}$} of each point in the original point set, while $\vec{x^{'}}$ is the transformed 3D position. A is a matrix to apply the random affine transformation. More specifically, A=I+U, where I is a 3 x 3 identity matrix, and U is a 3 x 3 random matrix with each element $U_{ij}$ from a uniform distribution. We used $U_{ij}\sim uniform\left(-0.05,0.05\right)$ in this study. $\vec{\varepsilon_{1}}$ is the 3D vector for adding random movements to each point in a point set, while $\vec{\varepsilon_{2}}$ is for adding even larger random movements in a subset of points (20 out of 175 cells) to simulate serious errors from segmentation procedures. We used $\varepsilon_{1,i}\sim uniform\left(-2,2\right)$ and $\varepsilon_{2,i}\sim uniform\left(-5,5\right)$ in this study. By randomly generating $U_{ij}$, $\varepsilon_{1,i}$ and $\varepsilon_{2,i}$, we could generate an arbitrarily large number of new point sets with new positions from the original point set. After that, we chose a specific point A and another point B from each generated point set and the original point set, respectively, and we calculated their relative positions as inputs for the FFN. In half of the cases, points A and B are corresponding, that is, they come from the same cell, while in the other half, point A is from a cell adjacent to the cell of point B, thus they are not corresponding. In this study, we used 576,000 newly generated pairs of points A and B for training the FFN (Figure 1C). We used binary cross-entropy as the loss function to train the FFN. During the training, the performance of matching by FFN was gradually improved using an independent test dataset of two point sets (Figure 2—figure supplement 2B).

### PR-GLS method

The initial match calculated using FFN was corrected using the expectation–maximization (EM) algorithm in the PR-GLS method, as described in the original paper (Ma et al., 2016). In the original study, the initial match (by FPFH) was recalculated during the EM iterations; however, in most datasets, we calculated the initial match only once (by the FFN) before the EM steps were performed, which did not cause problems. Only for the dataset worm #4 with very large movements, we recalculated initial matching by FFN after every 10 iterations, in order to improve accuracy. After the PR-GLS corrections, we obtained coherent transformations from the points of each volume to the subsequent volume (Figure 2C, panel 4–2).

### Single and ensemble modes

The FFN + PR-GLS can predict new cell positions at time *t* from the cell positions at *t*-1; this is the default mode of our pipeline, which is referred to as the single mode (Figure 6C). At a sufficiently high sampling rate, the single mode is reasonable because the movement from *t*-1 to *t* is much smaller than that from *t-i* (*i* > 1) to *t*, making the prediction from *t*-1 more reliable.

When cell movements are very large (such as in a freely moving worm), and the sampling rate is not sufficient, the prediction from *t*-1 to *t* becomes less reliable, and the average of multiple predictions from *t-i* to *t* may be more accurate. Therefore, we developed an approach using the average of multiple predictions, referred to as the ensemble mode (Figure 6C). We tested this mode only on the worm #4 dataset, which had quite large movements (Figures 6B and 10, and Table 3), because the runtime of the ensemble mode is much longer than that of the single mode, that is, the runtime of the FFN + PR-GLS component is proportional to the number of predictions used. Specifically, the runtime including segmentation and tracking for worm #4 was approximately 30 volume/h in the single mode and 6 volumes/h in the ensemble mode.

In the ensemble mode, we calculated an average of up to 20 predictions from previous time points. In cases for which t ≤ 20, the averages was calculated from time points [*t*-1, *t*-2, …, 1]; in cases for which t > 20, it was calculated over [*t-d*, *t*-2*d*, …, *t*-20*d*], where *d* is the quotient (*t*-1)//20.

### Accurate correction for tracking

By applying the PR-GLS method to the initial match, we obtained a more reliable transformation function in which all obvious incorrect matches were corrected. However, small differences still existed in a few cells, which could accumulate over time to become large differences without correction. Thus, we included an additional automatic correction step, in which the center position of each cell was moved slightly toward the centers of each 3D U-Net detected region (for details, see Figure 2—figure supplement 4). After correction, all cells were moved to the estimated positions with their shapes unchanged from volume #1. For most of the datasets, we applied only one correction; for the worm #4 and tumor spheroid datasets, we applied corrections up to 20 times, until achieving convergence. If multiple cells overlapped in the new positions, we applied the watershed method again to assign their boundaries. In carrying out this step, we calculated the images of the tracked cell regions based on an interpolation of the raw image from volume #1, when the resolution along the *z* axis was much lower than that in the *x-y* plane (i.e. all datasets except for the worm #4 and the zebrafish). We did this in order to obtain more accurate estimates for each cell region.

### Manual correction of segmentation

We manually corrected the segmentation only in volume #1. We superimposed the automatically segmented regions of the volume #1 3D image onto the raw 3D image in the ITK-SNAP software, and we discarded false positive regions, such as autofluorescence regions and neuronal processes. If any cells were not detected (false negative error), we reduced the noise level parameter in the pre-processing (Table 1) which can eliminate such errors, or we manually added these cells in ITK-SNAP. Oversegmentation and undersegmentation regions were corrected carefully by considering the sizes and shapes of the majority of cells. The overall error rates depend on the image quality, but we usually found that around 10% of cells required manual correction which usually took 2–3 hr (for 100–200 cells).

### Visual inspection of tracking results

We counted the tracking errors of all cells by visually inspecting the tracking results in each volume (Figure 1—figure supplement 2). To confirm the tracking of worm’s neurons, we combined two 3D + T images—the raw images and the tracked labels—in a top–bottom arrangement displayed as a hyperstack by the ImageJ software to compare the cell locations in each volume. As the cells in worms’ datasets primarily moved in the x-y plane (Figures 3F and 9L, and Figure 3—figure supplement 2F), we observed the correspondence between the raw images and the tracked labels in each x-y plane to identify tracking errors in the results. To confirm our tracking of the tumor spheroid dataset, we applied the same method although there were many small cross-layer movements in the images (Figure 8F and G).

It was more difficult to confirm the tracking results in the hyperstack images for the cells in the freely moving worm and the zebrafish heart than in the semi-immobilized worm and tumor spheroid, due to the frequent occurrence of large movements of cells across layers (Figures 6G and 7G). Therefore, we superimposed images of the tracked labels onto the raw images and imported them into IMARIS (Bitplane, UK), and then visually checked the tracking results of each cell individually in 3D mode. For the zebrafish dataset with repeated oscillations, we tracked and checked all 98 cells from 1000 volumes. Because the freely moving worm engaged in irregular movements in three directions, visual checking was more challenging, and thus we only tracked and checked the initial 500 volumes of 3D images out of 1519 original volumes (Figure 6F).

### Evaluating large movements

Large movements of cells is one issue that makes tracking challenging. To evaluate how challenging each cell movement is, we defined the 'relative movement' (*RM*) of cell A at time *t* as: *RM* = (movement of cell A from *t*-1 to *t*) / (distance between cell A and its closest neighboring cell at *t*).

Figure 4A middle and right panels illustrate two cells moving in one dimensional space with *RM* ≥0.5. In this condition, a very simple tracking method, ‘search for the closest cell at the next time point’ will mistakenly match the cell A at *t* = 2 to the cell B at *t* = 1. Therefore, we argue that movements with *RM* ≥0.5 are more challenging than movements with *RM* <0.5.

It should be noted that large movement is not the only challenge for tracking. For example, the zebrafish datasets have much higher error rates if we evaluate the tracking in all cells (Table 3), which is likely caused by the weak intensities in these small cells and the photobleaching (Figure 7B and F; Figure 7—figure supplement 1).

### Extracting activities

After having tracked the worm’s neurons from the first volume to the last volume, we extracted activities from the regions corresponding to each neuron. By measuring the mean intensities of each neuron in both channels corresponding to the GECI and the positional markers, the activity was computed as GCaMP5G/tdTomato in dataset worm #1. We similarly extracted calcium activities (GCaMP) of heart cells in the zebrafish dataset and the FRET activity in the tumor spheroid dataset.

### Comparison of tracking accuracy between our method and two other methods

Because DeepCell 2.0 currently only supports tracking cells in 2D datasets, we tested it using two layers of images (*z* = 9 and *z* = 16) from worm dataset #3, which include relatively large numbers of cells. We excluded cells that disappeared or appeared due to cross-layer movements for a fair comparison. We supplied DeepCell 2.0 with the precise segmentations from our method in order to focus on comparing the performance of the tracking algorithms.

We tested Toyoshima’s software using two worm datasets and one zebrafish dataset. For the zebrafish dataset, we only tested the initial 100 volumes because their method required a longer time for the imaging processing than ours did (see Table 5).

For these two methods, we communicated with the corresponding authors and did our best to optimize the parameters based on their suggestions. Nevertheless, it is possible that those analyses could be further optimized for our datasets.

### The runtimes of our pipeline and previous methods for the tested datasets

We tested the runtimes of our method and the previous methods for different datasets (see Table 5). Because DeepCell 2.0 currently can only track 2D + T images, the runtime was estimated by using the average runtime for one layer and multiplying that by 21 layers, in order to compare it with our method. As a result, DeepCell 2.0 required a runtime comparable with our method. On the other hand, Toyoshima’s software took a much longer time than our method to process the worm and zebrafish datasets.

In our method, the initial matching using our custom feedforward network is performed in a pairwise fashion, so the time complexity is *O(n^2^)*, where *n* is the number of detected cells. In our tested datasets with 100 ~ 200 cells, this did not take a long time, for example,~3.3 s were required for matching 98 cells between two volumes (zebrafish), or ~8.6 s for 164 cells (worm #1a) using our desktop PC. In cases where the *n* is too large, the runtime may become much longer, for example,~4 min for 901 cells (tumor spheroid). If both *n* and volume number are too large, it may be required to optimize the method to reduce the runtime, for example, by restricting the calculation of matchings in a set (100 ~ 200 or more) of representative cells (e.g. large/bright cells), while movements of other non-representative cells can be estimated from the movements of these representative cells utilizing the coherency of these movements in a deforming organ.

### Worm strains and cultivation

The techniques used to culture and handle *C. elegans* were essentially the same as those described previously (Brenner, 1974). Both TQ1101 *lite-1(xu7)* and AML14 were obtained from the Caenorhabditis Genetics Center (University of Minnesota, USA). Young adult hermaphrodites were used in the imaging experiments.

For pan-neuronal expression, *NLS::tdTomato::NLS* (Frøkjær-Jensen et al., 2016) and *NLS::GCaMP5G::NLS* (in which *GCaMP5G* (Akerboom et al., 2012) was codon-optimized for *C. elegans* and attached to *NLS* at N- and C-termini) were fused with the *rab-3* promoter (Stefanakis et al., 2015) using a GATEWAY system (Thermo Fisher Scientific). Germline transformation into *lite-1(xu7)* (Liu et al., 2010) was performed using microinjection (Mello et al., 1992) with a solution containing pYFU251 *rab-3p::NLS::GCaMP5G::NLS* (25 ng/µl), pYFU258 *rab-3p::NLS::tdTomato::NLS* (20 ng/µl) and OP50 genome (55 ng/µl) to obtain the strain KDK54165. The strain *lite-1(xu7)* was used to reduce the blue light-induced activation of the worm’s sensory system (Liu et al., 2010). Independent transgenic lines obtained from the injection produced similar results.

### Worm datasets

In this study, we used four worm datasets. The 3D images in datasets worm #1 and #2 were obtained using our custom-made microscope system, OSB3D (see below). The worm strains for datasets worm #1 and #2 are KDK54165 (RRID:WB-STRAIN:KDK54165) and AML14 *wtfEx4[rab-3p::NLS::GCaMP6s: rab-3p::NLS::tagRFP]* (RRID:WB-STRAIN:AML14) (Nguyen et al., 2016), respectively. The 3D + T images of the dataset worm #3 were published previously with the worm strain JN2101 *Is[H20p::NLS4::mCherry]; Ex[tax-4p::NLS-YC2.60, lin-44p::GFP]* (Toyoshima et al., 2016). The 3D + T images of the freely moving worm dataset (worm #4) were published previously with the worm strain AML14 (Nguyen et al., 2016; Nguyen et al., 2017). In three (#79, #135, and #406) out of the 500 volumes of the worm #4 dataset, most of the cells disappeared from the volume or a large amount of noise occurred, which rendered the dataset impossible to analyze. These volumes were therefore manually skipped for tracking, that is the cells were assumed not moved from the previous volumes.

### Spinning disk confocal system for 3D + T imaging of the worm's brain

We upgraded our robotic microscope system (Tanimoto et al., 2017) to a 3D version. We used a custom-made microscope system that integrated the Nikon Eclipse Ti-U inverted microscope system with an LV Focusing Module and a FN1 Epi-fl attachment (Flovel, Japan). The excitation light was a 488 nm laser from OBIS 488–60 LS (Coherent) that was introduced into a confocal unit with a filter wheel controller (CSU-X1 and CSU-X1CU, respectively; Yokogawa, Japan) to increase the rotation speed to 5,000 rpm. The CSU-X1 was equipped with a dichroic mirror (Di01-T-405/488/561, Semrock) to reflect the 488 nm light to an objective lens (CFI S Fluor 40X Oil, Nikon, Japan), which transmitted the GCaMP fluorescence used for calcium imaging and the red fluorescence used for cell positional markers. The laser power was set to 60 mW (100%). The fluorescence was introduced through the CSU-X1 into an image splitting optic (W-VIEW GEMINI, Hamamatsu, Japan) with a dichroic mirror (FF560-FDi01, Opto-line, Japan) and two bandpass filters (BA495-540 and BA570-625HQ, Olympus, Japan). The two fluorescent images were captured side-by-side on an sCMOS camera (ORCA Flash 4.0v3, Hamamatsu, Japan), which was controlled by a Precision T5810 (Dell) computer with 128 GB RAM using the HCImage Live software (Hamamatsu) for Windows 10 Pro. A series of images for one experiment (approximately 1–4 min) required approximately 4–15 GB of space, and were stored in memory during the experiment, then transferred to a 1-TB USB 3.0 external solid-state drive (TS1TESD400K, Transcend, Taiwan) for further processing.

For 3D imaging, the *z*-position of the objective lens was regulated by a piezo objective positioner (P-721) with a piezo controller (E665) and the PIMikroMove software (PI, Germany). The timings of the piezo movement and the image capture were regulated by synchronized external edge triggers from an Arduino Uno (Arduino, Italy) using 35 ms intervals for each step, in which the image capture was 29.9 ms. For each step, the piezo moved 1.5 µm, and one cycle consisted of 29 steps. We discarded the top-most step because it frequently deviated from the correct position, and we used the remaining 28 steps. Note that one 3D image was 42 µm in length along the *z*-axis, which was determined based on the typical diameters of neuronal cell bodies (2–3 µm) and of a young adult worm’s body (30–40 µm). Each cycle required 1015 ms; thus, one 3D image was obtained per second. This condition was reasonable for monitoring neuronal activities because the worm’s neurons do not generate action potentials (Goodman et al., 1998) and because many neuronal responses change on the order of seconds (Nichols et al., 2017). We also tested a condition using 10 ms for each step and 4.9 ms for an exposure with the same step size and step number per cycle (i.e. 2.3 volumes of 3D images per second), which yielded a comparable result. For cyclic regulation of the piezo position, we used a sawtooth wave instead of a triangle wave to assign positional information because the sawtooth wave produced more accurate *z* positions with less variance between cycles.

### Zebrafish heart cells

Sample preparation and imaging have been described previously (Voleti et al., 2019). In brief, GCaMP and dsRed were expressed in the cytosol and the nuclei of myocardial cells, respectively. The 3D + T images obtained with the SCAPE 2.0 system were skew corrected to account for the oblique imaging geometry and analyzed with 3DeeCellTracker. The 3D coordinates obtained from this study were used to extract calcium dynamics in the cells in the previous study (Voleti et al., 2019).

### Tumor spheroid

The tumor spheroid was cultured according to the previous procedures (Vinci et al., 2012; Yamaguchi et al., 2021). In brief, a suspension of HeLa cells (RRID:CVCL_0030, RCB0007, Riken Cell Bank; certified as mycoplasma-free and authenticated by using STR profiling) expressing the FRET-type ERK sensor EKAREV-NLS (Komatsu et al., 2011) was added to a PrimeSurface 96 well plate (MS-9096U, Sumitomo Bakelite) for 3D cell culture at a density of 1200 cells/well. The grown spheroids were transferred to 35 mm-dishes coated with poly-L-lysine (Sigma-Aldrich) and further grown with DMEM/F-12, no phenol red (ThermoFisher) containing 10% FBS and 1% penicillin and streptomycin.

The 3D + T images of the spheroid were recorded using a two-photon microscope equipped with a water-immersion objective lens (N25X-APO-MP 25x CFI APO LWD objective, Nikon) and a high-sensitivity gallium arsenide phosphide (GaAsP) detector (A1R-MP+, Nikon). An 820 nm optical pulse generated by a Ti:Sapphire laser was used for the excitation. The fluorescence was split using two dichroic mirrors (FF495-Di03 and FF593-Di02, Opto-line), and the split lights below 495 nm and between 495–593 nm were detected by independent channels (CH1 and CH2, respectively). The fluorescence signals were integrated four times to increase the signal-to-noise ratio. Each step size was 4 µm along the *z-*axis, and each volume comprised of 54 steps, requiring 5 min for recording. Cells that experienced cell death or cell division were excluded manually after the events when evaluating the tracking accuracy.

### Code availability statement

The code for tracking cells and for training neural networks, the demo data, the pre-trained weights of the neural networks, and instructions for installation and use of the code are available in http://ssbd.qbic.riken.jp/set/20190602/ (Demos190610.zip). An updated version of the code can be found in https://github.com/WenChentao/3DeeCellTracker. The guides for using the codes and for setting parameters have also been included in the same GitHub repository.

## Note in proof

In the original version of the paper, we tracked the datasets by our original version of programs based on the package "3DeeCellTracker 0.2" (see the version information at https://pypi.org/project/3DeeCellTracker/#history). The runtime for the tracking process was not optimized at that time, which sometimes could lead to a long runtime for tracking, especially in the ensemble mode and/or when the cell number is large. In addition, our previous tracking program did not hide the details irrelevant to the end-users and did not provide useful feedback on the intermediate segmentation/tracking results to users.

To solve these two issues, we first improved the performance of the codes related to “FFN” and “PR-GLS” using the vectorization techniques available in the “NumPy” package in Python. We also improved the performance of the codes related to “accurate correction” by extracting and performing only once the previously repeated calculations in the loops. Note that we did not change our programs related to segmentation (3D U-Net + watershed), which may still require considerable time depending on factors such as the image size and structure of the 3D U-Net. As a result, our new program accelerated the speed by 1.7–6.8 times in the tested datasets (Table 6). Such acceleration was especially pronounced in ensemble mode (worm #4) and when the cell number is large (3D tumor spheroid). Second, we simplified the program by moving the details into the package. As a result, users can track cells by simply running a few commands in Jupyter notebooks. We also added new functions to show the intermediate results of segmentation and tracking so that users can now use these results to guide their setup of the segmentation/tracking parameters. To use these new features, users can follow the updated instructions in the README file in our GitHub repository to install the latest version of 3DeeCellTracker (currently 0.4.0) and use our Jupyter notebooks.

**Table 6.** Runtime and speed of our programs, including both the segmentation and tracking. The speeds were calculated as the reciprocals of runtimes.

	Runtime (s/volume)		Speed (volume/h)	
	Current (0.4.0)	Previous (0.2)	Current (0.4.0)	Previous (0.2)
Worm #1a	21	106	171	34
Worm #3	23	38	156	95
Worm #4 (single)	73	122	49	30
Worm #4 (ensemble)	86	589	42	6
Zebrafish	21	60	171	60
﻿Tumor spheroid	128	834	28	4

## Data Availability

Datasets of worm #1a, #1b, and #2, and the tumor spheroid (referred to in Figure 3, Figure 3-figure supplement 1 and 2, and Figure 8, respectively), consisting of nuclear and calcium images are available in http://ssbd.qbic.riken.jp/set/20190602/. The nuclear and calcium images of dataset worm #3 (Figure 3-figure supplement 3) have been described in the previous study (Toyoshima et al., 2016) and are available in http://ssbd.qbic.riken.jp/search/D4D6ACB6-3CF4-11E6-B957-D3D8A1F734EE/. The freely moving worm dataset worm#4 has been described in the previous study (Nguyen et al. 2017) and is available in: https://ieee-dataport.org/open-access/tracking-neurons-moving-and-deforming-brain-dataset. The nuclear and calcium images of the zebrafish dataset (Figure 7) are available upon request according to the previous study (Voleti et al., 2019). The following dataset was generated: WenCMiuraTVoletiVYamaguchiKTsutsumiMYamamotoKOtomoKFujieYTeramotoTIshiharaTAokiKNemotoTHillmanEMCKimuraKD2021Time-lapse 3D imaging data of cell nucleiSystems Science of Biological Dynamics databasehttp://ssbd.qbic.riken.jp/set/20190602/ The following previously published datasets were used: ToyoshimaYTokunagaTHiroseOKanamoriMTeramotoTJangMSKugeSIshiharaTYoshidaRIinoY2016BDML file for quantitative information about neuronal nuclei in C. elegans (strain JN2101) adult extracted from time-lapse 3D multi-channel spinning disk confocal microscopy (SDCM) imagesSystems Science of Biological Dynamics databasehttp://ssbd.qbic.riken.jp/search/D4D6ACB6-3CF4-11E6-B957-D3D8A1F734EE/ NguyenJPLinderANPlummerGSShaevitzJWLeiferAM2017TRACKING NEURONS IN A MOVING AND DEFORMING BRAIN DATASETIEEE DataportDOI:10.21227/H2901H10.1371/journal.pcbi.1005517PMC543663728545068

## Additional files

Transparent reporting form
