# Supplementary material for: 3DeeCellTracker, a deep learning-based pipeline for segmenting and tracking cells in 3D time lapse images
Source: eLife. 2021 Mar 30;10:e59187. doi: 10.7554/eLife.59187 (PMC8009680; doi:10.7554/eLife.59187)
Supplement: Peer review history

# Decision letter

**DOI:** 10.7554/eLife.59187.sa1

**Affiliations:** Research Institute of Molecular Pathology, Vienna Biocenter and University of ViennaAustria

In the interests of transparency, eLife publishes the most substantive revision requests and the accompanying author responses.

**Acceptance summary:**

This paper is of great interest to system-biologists who aim to analyze large datasets of image-volume time series that contain objects to segment in continuously deforming life tissues. Wen et al. present a deep-learning method for mostly automatic 1) U-net based segmentation and 2) point-registration-based tracking of cells in tissues with ongoing deformations. The authors test their pipeline on various example systems: whole brain Ca^2+^-activity in partially immobilized and freely crawling *C. elegans*, beating zebrafish heart and a 3D culture of tumor tissue. This work presents significant improvements in tracking capabilities on various metrics including the possible number of tracked objects, robustness, and computing requirements.

**Decision letter after peer review:**

Thank you for submitting your article "3DeeCellTracker, a deep learning-based pipeline for segmenting and tracking cells in deforming organs" for consideration by *eLife*. Your article has been reviewed by two peer reviewers, one of whom is a member of our Board of Reviewing Editors, and the evaluation has been overseen by Ronald Calabrese as the Senior Editor. The reviewers have opted to remain anonymous.

The reviewers have discussed the reviews with one another and the Reviewing Editor has drafted this decision to help you prepare a revised submission.

As the editors have judged that your manuscript is of interest, but as described below that additional experiments are required before it is published, we would like to draw your attention to changes in our revision policy that we have made in response to COVID-19 (https://elifesciences.org/articles/57162). First, because many researchers have temporarily lost access to the labs, we will give authors as much time as they need to submit revised manuscripts. We are also offering, if you choose, to post the manuscript to bioRxiv (if it is not already there) along with this decision letter and a formal designation that the manuscript is "in revision at *eLife*". Please let us know if you would like to pursue this option. (If your work is more suitable for medRxiv, you will need to post the preprint yourself, as the mechanisms for us to do so are still in development.)

Summary:

This paper presents a deep-learning method for mostly automatic 1) U-net based segmentation and 2) point-registration-based tracking of cells with some deformations. Their training data consists of a single volume of corrected annotated data. The authors test their pipeline on two example systems: 3 datasets of ~150 neurons each in partially immobilized *C. elegans* and ~100 cells in a beating zebrafish heart. Some datasets are from previous work [1,2] and some are newly acquired. Both of these previous works propose tracking pipelines, and this work claims improvements in tracking capabilities on various metrics including number of segmented neurons, percentage of correct identifications, and runtime. In addition, the authors degrade their datasets in various ways (adding noise, removing frames), and still retain good performance. However, both of these claims lack proper quantifications.

The general idea of using deep learning for segmentation, and for tracking, is timely. However, the manuscript feels at times anecdotal and it is not convincing that it is truly ready for the diverse applications it claims. The consensus among the reviewers is that the approach should be tested on more ground truth data-sets, which are available for worms and on repeated measurements from zebrafish heart. Moreover, it is unclear how well the approach would work from truly deforming tissues like unrestrained moving worms. Such datasets are available from the Leifer lab as well as from the Hillman lab, who is co-authoring this manuscript. Claims on the performance of their approach should be based on more solid quantifications.

Essential revisions:

*Reviewer #1:*

1) Motion in data

a) This pipeline would be a major breakthrough if tracking succeeded in the freely moving scenario across different species, as in previous custom-made trackers for *C. elegans* [3]. Indeed, this is the core challenge in video processing of calcium imaging data, as classical algorithms largely suffice for the no motion or slow-motion cases. To my understanding Nguyen et al., 2016, was from freely moving worms but the datasets used here seems all from an immobilized worm (worm #1-2). Please clarify. A better description of the used datasets should be provided instead of simply citing the original sources.

b) However, it is unclear how much of a contribution this pipeline is in this respect. While the zebrafish heart data are clearly moving and deforming (Figure 5—video 2), the motion is extremely stereotyped. The *C. elegans* videos however, do not appear to be moving (Figure 3—videos 1-2, Figure 4—videos 1-2). If this algorithm does not work with the motion present in freely moving animals, this should be clarified.

c) If it does, more explicit comparisons with the state of the art are required, e.g. [3].

d) Regardless of the exact claim, some quantification of tracking performance as a function of increased motion should be included. Although robustness tests are performed by removing intermediate volumes from the videos, due to the nature of the motion (cyclic in zebrafish and intermittent in *C. elegans*), it is not obvious if or in what way this increases the motion of the neurons

2) Feed-Forward Network (FFN) for initial tracking

a) This network is the sole deep-learning component of the second stage of the algorithm, finding correspondence between segmentations in different volumes. However, it is unclear what benefit this element brings, and the statement starting on "We believe that the high accuracy and robustness of our method is based on the introduction of FFN for tracking" seems unwarranted.

b) In particular: the FFN is trained on random affine transformations of segmented volumes (Materials and methods); thus, it is surprising that it would perform better than a direct affine alignment.

c) This comparison should be included, and if the FFN is better, some discussion motivating this surprising result should also be included.

3) Figure 2, part 2:

Panels A and B show FPFH and FNN performance (respectively) on a matching task, but neither appears to be the algorithm used in the actual pipeline, which is a combination of both. In addition, why is the matching shown between t=1 and t=10? I agree with the caption that this should be more challenging for the algorithm, but without the context of t=1 -> t=2 or further quantification, it is hard to understand the goal of these panels.

This figure could be significantly improved by adding quantifications due to robustness to motion as discussed above.

4) Figure 5:

Photobleaching is mentioned as causing difficulties with segmentation, but this is not quantified.

5) Related to usability: A key claim of this paper is that the authors' package is easy to use. Indeed, I applaud the use of github and freely available software like Tensorflow. This claim is generally supported by 1) the fact that the work runs on a desktop (favorably comparing to other trackers, e.g., which require a cluster [3]) and 2) the multiple example systems and optical systems tested on. However, there are several key concerns:

a) Basic automatic conda environment creation should be supported; see the.yaml files in e.g. DeepLabCut and the associated tutorial

https://github.com/DeepLabCut/DeepLabCut/blob/master/conda-environments/DLC-CPU.yaml

b) Why is Fiji a dependency? Although I personally love Fiji, simple rigid alignment can be performed in python and would remove a fragile dependency

c) What implementation of PR-GLS is being used? The original publication references a MATLAB implementation: https://github.com/jiayi-ma/PR-GLS. However, the authors are using entirely Python… did the authors rewrite the original algorithm? Or is there another implementation not associated with the paper? This should be clarified

*Reviewer #2:*

1) The work uses way too few sets of validation data. Altogether, it only used three sets of *C. elegans* wholebrain imaging data and a single set of zebrafish heart imaging data. Drawing general conclusions from this on the algorithm's performance (especially comparisons with other algorithms) is premature.

2) In the comparisons with other methods, while the intention was good, the results are anecdotal. Testing the algorithms is done on a very limiting set of data; whether these datasets represent the breadth of realistic problems that would be encountered for the variety of applications (e.g. neuroscience, or other physiological or developmental functional imaging, in *C. elegans* or zebrafish or other model systems) is hard to tell. Also quite importantly, it is difficult to say whether the optimal choice of parameters were used for the competing methods.

3) Noises added to the real data (as in Figure 6) aren't necessarily representative of the real data's properties/features. There should be characterizations of the variability in real data on size, intensity, shape, etc. Further, it does seem that the performance of the algorithm is sensitive to these noises, and thus it is not trivial to prove that the algorithm is robust to these noises. The manuscript did not convince me that the algorithm currently can indeed deal with real noises in real datasets.

4) "To solve the difficulty in preparing training data for FFN, we used artificial training data by simulating cell movements in deforming organs." Why is this good? How is the artificial data representing real organ deformation?

5) Results, comparing other datasets – the comparisons are very qualitative. Validation is vague, descriptive, rather than quantitative.

6) Several places of the manuscript are also anecdotal, e.g. Results (corrupt data, still get 53% correct). Even the in silico studies (e.g. noise or reduced sampling) aren't systematic.

7) 60-80% of zebrafish heart cells tracked – is it useful? Which ones are the missing cells? The qualitative conclusions seem to be fairly trivial.

The manuscript made a few assertions without backing up with data/results. For example, the problems identified are real (Results), but no specifics to back up, and no systematic characterizations are done, using real data, comparing the work with other approaches. Another example – "our method became robust to tracking errors in the initial matching".

References:

1) "Accurate Automatic Detection of Densely Distributed.… – PLoS." 6 Jun. 2016, https://journals.plos.org/ploscompbiol/article?id=10.1371/journal.pcbi.1004970. Accessed 26 Jun. 2020.

2) "Real-time volumetric microscopy of in vivo dynamics and large.…." 27 Sep. 2019, https://www.nature.com/articles/s41592-019-0579-4. Accessed 26 Jun. 2020.

3) "Automatically tracking neurons in a moving and.… – PLoS." 18 May. 2017, https://journals.plos.org/ploscompbiol/article?id=10.1371/journal.pcbi.1005517. Accessed 26 Jun. 2020.

# Reply: Author response

**DOI:** 10.7554/eLife.59187.sa2

Summary:

This paper presents a deep-learning method for mostly automatic 1) U-net based segmentation and 2) point-registration-based tracking of cells with some deformations. Their training data consists of a single volume of corrected annotated data. The authors test their pipeline on two example systems: 3 datasets of ~150 neurons each in partially immobilized *C. elegans* and ~100 cells in a beating zebrafish heart. Some datasets are from previous work [1,2] and some are newly acquired. Both of these previous works propose tracking pipelines, and this work claims improvements in tracking capabilities on various metrics including number of segmented neurons, percentage of correct identifications, and runtime. In addition, the authors degrade their datasets in various ways (adding noise, removing frames), and still retain good performance. However, both of these claims lack proper quantifications.

The general idea of using deep learning for segmentation, and for tracking, is timely. However, the manuscript feels at times anecdotal and it is not convincing that it is truly ready for the diverse applications it claims. The consensus among the reviewers is that the approach should be tested on more ground truth data-sets, which are available for worms and on repeated measurements from zebrafish heart. Moreover, it is unclear how well the approach would work from truly deforming tissues like unrestrained moving worms. Such datasets are available from the Leifer lab as well as from the Hillman lab, who is co-authoring this manuscript. Claims on the performance of their approach should be based on more solid quantifications.

First of all, we would like to thank the editor and the reviewers for their positive and constructive comments on our original manuscript. To address the comments, we have performed the following:

1) We have tested two additional types of ground truth datasets. The first is a previously published dataset of a freely moving worm from the Leifer lab, from which we were able to track 99.8% of all cell movements by developing a new "ensemble mode". The second is a 3D culture of ~900 tumor cell dataset, which was newly obtained for this study to demonstrate the applicability of our method for general biomedical research; the registration of many cell positions in a 3D culture to monitor their activities has become one of the crucial issues in recent microscopy-based life science research and in drug discovery. Because of these data additions, we modified the manuscript title partially. Additional zebrafish data was not obtained unfortunately under the current difficult quarantine conditions, and the neuronal images of a freely moving worm from the SCAPE system does not have sufficient resolution to segment currently for our method.

2) For most of the figures, we have added solid quantifications, such as cell intensities, cell movements, accuracy curves and positions of correctly/incorrectly tracked cells in the tracking results, etc.

3) We have also deleted as much anecdotal phrases as possible from the entire text and toned down the discussions on diverse applications.

Essential revisions:

Reviewer #1:

1) Motion in data

a) This pipeline would be a major breakthrough if tracking succeeded in the freely moving scenario across different species, as in previous custom-made trackers for *C. elegans* [3]. Indeed, this is the core challenge in video processing of calcium imaging data, as classical algorithms largely suffice for the no motion or slow-motion cases. To my understanding Nguyen et al., 2016, was from freely moving worms but the datasets used here seems all from an immobilized worm (worm #1-2). Please clarify. A better description of the used datasets should be provided instead of simply citing the original sources.

We apologize for the unclear description in the original manuscript. In the original manuscript, we used our own microscope system to image neurons in anesthetized AML14, the same strain used in Nguyen et al., 2016 (worm #2). We have clarified the experimental conditions of AML14 and other worms in the main text.

In addition, in this revised version, we analyze the freely moving worm dataset previously published by Nguyen et al., and describe how we were able to track most of the cell movements. For details, please see below and Figure 6. We appreciate the reviewer's comment, which has motivated us to track the neurons of a freely moving worm.

b) However, it is unclear how much of a contribution this pipeline is in this respect. While the zebrafish heart data are clearly moving and deforming (Figure 5—video 2), the motion is extremely stereotyped. The *C. elegans* videos however, do not appear to be moving (Figure 3—videos 1-2, Figure 4—videos 1-2). If this algorithm does not work with the motion present in freely moving animals, this should be clarified.

We showed (1) that the deep learning-based DeepCell 2.0 tracked ~10% of neurons in a semi-immobilized worm, whereas our method achieved 95–100% tracking (Figure 11—figure supplement 1A, Figure 11—video 1 and Table 4), and (2) that Toyoshima's method was able to track only ~40% of the zebrafish heart cells that our method could track (35 versus 84; Figure 11—figure supplement 1B and Table 4) with a run time that was 15 times longer than ours (Table 5). In addition, because our original program ("single mode"; see below) predicts cell positions from the previous volume, whether the motion is stereotyped (e.g., zebrafish heart) or not does not affect the tracking efficiency. We consider that these results demonstrate the advantages of our method to the previous methods even with the original version ("single mode"). Furthermore, we show that our program is now able to track the neurons of a freely moving worm with "ensemble mode" (please see next).

c) If it does, more explicit comparisons with the state of the art are required, e.g. [3].

Our original method ("single mode") was able to track 73% of the cells, which was acceptable but not ideal. Therefore, we developed a new "ensemble mode". To obtain robust tracking in the ensemble mode, we use the FFN to predict cell positions based on a calculation of the average of multiple predictions from different volumes (i.e. time points). Our method with ensemble mode was able to correctly track 99.8% of cell movements. Unfortunately, we were not able to find a corresponding description of the accuracy in the Nguyen et al., and therefore were unable to explicitly compare our outcomes. Nevertheless, we consider that our obtained 99.8% accuracy is sufficient. In addition, we would like to note that, while the method by Nguyen et al. requires a high-performance computing cluster, our method runs on a desktop PC with a GPU, which is more accessible to general researchers (Figure 6 and Results, Discussion and Materials and methods).

d) Regardless of the exact claim, some quantification of tracking performance as a function of increased motion should be included. Although robustness tests are performed by removing intermediate volumes from the videos, due to the nature of the motion (cyclic in zebrafish and intermittent in *C. elegans*), it is not obvious if or in what way this increases the motion of the neurons

We appreciate the comment. We have added graphs demonstrating the relationships between motion and error rates. Please refer to Figure 10—figure supplement 1 and 2.

2) Feed-Forward Network (FFN) for initial tracking

a) This network is the sole deep-learning component of the second stage of the algorithm, finding correspondence between segmentations in different volumes. However, it is unclear what benefit this element brings, and the statement starting on "We believe that the high accuracy and robustness of our method is based on the introduction of FFN for tracking" seems unwarranted.

We apologize for the unclear description and the absence of quantitative information in the original manuscript. We have quantified the differences in tracking accuracies obtained by our FFN in combination with the point set registration method (FFN + PR-GLS), the previous FPFH + PR-GLS, and the affine alignment (Figure 2—figure supplement 3C and D).

b) In particular: the FFN is trained on random affine transformations of segmented volumes (Materials and methods); thus, it is surprising that it would perform better than a direct affine alignment.

Again, we apologize for the unclear description in the original manuscript. Our method also adds random displacements for each cell independently after affine transformation. Thus, the simulated motions are not simply affine transformation but also include more complex deformations (Figure 2—figure supplement 2A and B).

c) This comparison should be included, and if the FFN is better, some discussion motivating this surprising result should also be included.

Affine alignment (used CPD algorithm from https://github.com/siavashk/pycpd) generated poorer predictions of cell positions than our FFN + PR-GLS. Please see Figure 2—figure supplement 3C and D. We appreciate the reviewer for having motivated us to compare our method with the affine alignment method.

3) Figure 2, part 2:

Panels A and B show FPFH and FNN performance (respectively) on a matching task, but neither appears to be the algorithm used in the actual pipeline, which is a combination of both. In addition, why is the matching shown between t=1 and t=10? I agree with the caption that this should be more challenging for the algorithm, but without the context of t=1 -> t=2 or further quantification, it is hard to understand the goal of these panels.

This figure could be significantly improved by adding quantifications due to robustness to motion as discussed above.

We apologize for the confusion caused by the description in the original manuscript. The panels A and B in Figure 2—figure supplement 3 are to compare the results of initial matching by FPFH and FFN. After the initial matching by the FPFH (Ma et al.) or by the FFN (our method), the point set registration method (PR-GLS) was used in both of the methods. To clarify this point and to demonstrate the differences after the matching, we added panels C and D in Figure 2—figure supplement 3.

We compared volumes #1 (*t*1) and #10 (*t*2) as an example of a challenging condition. We chose this comparison because its average value of relative movements (*RM*) of all the cells between volumes #1 and #10 is 1.22, while between volumes #1 and #2 the average *RM* is 0.08, indicating that the cell movements between volumes #1 and #2 are smaller and easier.

4) Figure 5:

Photobleaching is mentioned as causing difficulties with segmentation, but this is not quantified.

Thank you for pointing this out. We have added panels for the quantification of time course changes in the intensities of cell regions and backgrounds, some of which showed photobleaching (panel B middle in Figures 3, 6, 7, and 8). As shown in Figure 7B top and middle, the intensities of the small cells substantially overlap with background regions, and the overlap became even larger through time because of the photobleaching, causing difficulties to segment these small cells.

5) Related to usability: A key claim of this paper is that the authors' package is easy to use. Indeed, I applaud the use of github and freely available software like Tensorflow. This claim is generally supported by 1) the fact that the work runs on a desktop (favorably comparing to other trackers, e.g., which require a cluster [3]) and 2) the multiple example systems and optical systems tested on.

To clarify the point (2), we have added information about the microscope system to panel A of Figures 3, 6, 7, and 8.

However, there are several key concerns:

a) Basic automatic conda environment creation should be supported; see the.yaml files in e.g. DeepLabCut and the associated tutorial

https://github.com/DeepLabCut/DeepLabCut/blob/master/conda-environments/DLC-CPU.yaml

Thank you for the suggestion. We have added the “3DCT.yml” file and the related instructions in the “README.md” file in our “3DeeCellTracker” repository in GitHub to help users to create the same conda environment that we have used.

b) Why is Fiji a dependency? Although I personally love Fiji, simple rigid alignment can be performed in python and would remove a fragile dependency

The rigid alignment is not the core of our study. We only applied the alignment in worm #1 and #2 but not in other datasets. This step is not always required, and we did not include this step into our program. If users would prefer, this step can be easily replaced with a function written in Python. (Materials and methods)

c) What implementation of PR-GLS is being used? The original publication references a MATLAB implementation: https://github.com/jiayi-ma/PR-GLS. However, the authors are using entirely Python… did the authors rewrite the original algorithm? Or is there another implementation not associated with the paper? This should be clarified

We wrote the algorithm by ourselves with Python (see the “pr_gls” function in our “3DeeCellTracker” repository in GitHub) because the implementation had not yet been released when we were building our pipeline.

Reviewer #2:

1) The work uses way too few sets of validation data. Altogether, it only used three sets of *C. elegans* wholebrain imaging data and a single set of zebrafish heart imaging data. Drawing general conclusions from this on the algorithm's performance (especially comparisons with other algorithms) is premature.

In the revised version, we analyzed a dataset of a freely moving worm as suggested by reviewer #1 (Figure 6). In addition, we performed the registration of ~900 tumor cells in a 3D culture and measured ERK (=MAPK) activities, which are related to broader applications in biomedical research (Figure 8). In both of these cases, our pipeline achieved ≥ 97% of tracking. Additional zebrafish data was unfortunately not obtained under the current difficult quarantine conditions.

2) In the comparisons with other methods, while the intention was good, the results are anecdotal.

We appreciate the comment. We have added tracking accuracy curves for quantifying the comparison results (Figure 11—figure supplement 1).

Testing the algorithms is done on a very limiting set of data; whether these datasets represent the breadth of realistic problems that would be encountered for the variety of applications (e.g. neuroscience, or other physiological or developmental functional imaging, in *C. elegans* or zebrafish or other model systems) is hard to tell.

As noted above, we have added the analyses of a freely moving worm (Figure 6) and the ~900 tumor cells (Figure 8) throughout the manuscript. We also toned down the discussions on diverse applications.

Also quite importantly, it is difficult to say whether the optimal choice of parameters were used for the competing methods.

Prior to the initial submission, we had contacted the corresponding authors of the two methods, and optimized the parameters as much as possible according to their suggestions. However, it is still possible that their method could be further improved by applying unused parameter sets; we describe this possibility in the text. We have also modified the corresponding sentences to be more objective.

3) Noises added to the real data (as in Figure 6) aren't necessarily representative of the real data's properties/features. There should be characterizations of the variability in real data on size, intensity, shape, etc. Further, it does seem that the performance of the algorithm is sensitive to these noises, and thus it is not trivial to prove that the algorithm is robust to these noises. The manuscript did not convince me that the algorithm currently can indeed deal with real noises in real datasets.

We appreciate the comment. In this revision, we have described the overall distributions and time course changes in the cell/background intensities and the relative movements of cells (panel B in Figures 3, 6, 7, and 8) as well as the cell diameters, intensity and cell speeds (panel A in the same figures), all of which are from real datasets.

The background signal is particularly high in the zebrafish dataset (Figure 7B): even in the larger and higher-intensity cells, a portion of the cell signals overlapped with the background. If cell signal overlaps with background signal, even researchers will not be able to properly segment the cell, thus this is a very challenging condition. We achieved 87% tracking of the large and high intensity cells in all the 1000 volumes (Results). Moreover, in the first 100 volumes, our method tracked 86% of all the cells detected, which was considerably better than a previous method did (Toyoshima et al., 2016; Figure 11—figure supplement 1B and Table 4). It should be noted that the high background signal was caused by the extremely high speed image acquisition rate of the SCAPE system (10,568 fps); in all the other conditions on real datasets (≤ 100-200 fps), the cell signals were well separated from the background signals, and our method demonstrated ≥ 97% tracking efficiency.

4) "To solve the difficulty in preparing training data for FFN, we used artificial training data by simulating cell movements in deforming organs." Why is this good? How is the artificial data representing real organ deformation?

As mentioned in the response to the question 2b of reviewer #1, deep learning had not been previously used for 3D cell tracking because of the difficulty in preparing large amounts of training data. Therefore, we needed to develop a new technique to solve this problem, and decided to prepare artificial training data by simulating real cell movement. We used affine transformation to simulate the global coherent motions of cells (neighboring cells with similar movements), and added independent random movements to each cell to simulate local incoherent motions of cells (Figure 2—figure supplement 2). As a result, our method (FFN + PR-GLS) exhibited superior tracking results to the previous method (FPFH + PR-GLS) and to the affine alignment (Figure 2—figure supplement 3). Thus we consider that this result indicates that the use of artificially generated datasets for training is an effective method of employing deep network for cell tracking. (Results, Discussion and Materials and methods).

5) Results, comparing other datasets – the comparisons are very qualitative. Validation is vague, descriptive, rather than quantitative.

We apologize for the inappropriate description in the original manuscript. The tracking accuracy of worm #2 was also 100%. We have also added tracking accuracy curves for all the datasets (panel E in Figures 3 (and its figure supplements), 6 and 7; panel D in Figure 8; and panels D and J in Figure 9). Validation was based on visual inspection according to previous studies (for example Nguyen et al., 2017). We have added the details of our visual inspection in Figure 1—figure supplement 2 and in the Materials and methods.

6) Several places of the manuscript are also anecdotal, e.g. Results (corrupt data, still get 53% correct). Even the in silico studies (e.g. noise or reduced sampling) aren't systematic.

As mentioned above, we have added tracking accuracy curves for all datasets. We would like to note that this particular experiment (the zebrafish data on a reduced z-axis) was performed according to a suggestion by one of the reviewers for a previous submission to a different journal. We also described the reasons for the degenerate dataset analysis (Results).

7) 60-80% of zebrafish heart cells tracked – is it useful? Which ones are the missing cells? The qualitative conclusions seem to be fairly trivial.

We consider 60-80% of cell tracking is still informative because the tracked cells allow analysis of the relationships between calcium dynamics and the natural heartbeats in vivo (Figure 7H and I). This has finally become available due to the developments of a state-of-the-art SCAPE 2.0 system that can monitor 100 volumes per second, and of our software pipeline that can correctly track a large portion of these cell movements in 3D space (Results). Also, we have added the spatial localizations of the cells with correct/incorrect tracking (panel F in Figure 6 and 7; panel E in Figure 8; and panels E and K in Figure 9) for the datasets that did not have 100% accurate tracking.

The manuscript made a few assertions without backing up with data/results. For example, the problems identified are real (Results), but no specifics to back up, and no systematic characterizations are done, using real data, comparing the work with other approaches. Another example – "our method became robust to tracking errors in the initial matching".

We appreciate the comment. For the corresponding sentences, we have clarified our solutions to the problems and described quantitative comparisons with the previous methods: namely, we changed the sentence (Results) and added quantitative descriptions and detailed explanations (Figure 2—figure supplements 3 and 4).
